# Supplementary material for: Integrated Child and Family Hub models for detecting and responding to family adversity: protocol for a mixed-methods evaluation in two sites
Source: BMJ Open. 2022 May 19;12(5):e055431. doi: 10.1136/bmjopen-2021-055431 (PMC9125738; doi:10.1136/bmjopen-2021-055431)
Supplement: Supplementary data [file bmjopen-2021-055431supp001.pdf]

CAREGIVER SURVEY

2-8 Year Old Child

ABOUT YOU AND YOUR FAMILY

Are you the main caregiver to any child aged 0-8 years or currently pregnant? Yes ☐ No ☐

How many children live in your household? ___________

Do you have any children with a disability? Yes ☐ No ☐

Do you live with another adult such as a partner or other relative? Yes ☐ No ☐

If yes, who do you live with? (Select all that apply)

☐ Partner/husband/wife ☐ Grown up children ☐ Other, please specify

☐ Parents or in-laws ☐ Other relatives

What is your year of birth? ___________

What is your gender? (Pick which best applies)

☐ Woman ☐ Non-binary/gender diverse ☐ Prefer not to say

☐ Man ☐ My gender identity isn't listed, please specify

Do you identify as Aboriginal and/or Torres Strait Islander? Yes ☐ No ☐

Where were you born? Australia ☐ Other, please specify which country ☐ ___________

What language do you mainly speak at home? English ☐ Other, please specify ☐ ___________

What was the highest year of primary or secondary school you completed? (e.g. grade 6, year 10)

____

What is your postcode? ___________

Hall T, *et al. BMJ Open* 2022; 12:e055431. doi: 10.1136/bmjopen-2021-055431

CHALLENGES IN LIFE

In the next three sections, there are three lists of challenges that can make it difficult for families to thrive. We will ask you *how many* of these challenges have affected your family. You do not need to tell us *which challenges*.

If you have a pen handy, it may help you to make a mark on a piece of paper for each challenge that applies to you. You can then count these up at the end. You can also count on your hands.

LIST ONE: OUTSIDE OF THE FAMILY

Over the past 6 months, how many of the following *four* challenges have you felt concerned about affecting your child/ren or family:

- Not enough contact with or support from others for yourself
- Not enough money for everyday things such as food, clothing or bills
- Problems with housing like worrying about keeping your home, having to share your home, or having a house that's too crowded, or in need of repair

☐ 1 ☐ 2 ☐ 3 ☐ 4

Thinking about the *four* challenges we just spoke about, regardless of whether or not you have been concerned about them:

In the past 6 months, has any staff member at IPC Health asked you about any of these challenges?

Yes ☐ No ☐ I don't remember ☐

If yes:

Which staff member(s)? (select all that apply)

☐ Paediatricians
☐ GPs
☐ Maternal Child Health Nurses
☐ Other Nurses
☐ Lawyers
☐ Speech Pathologists
☐ Family Violence Workers
☐ Wellbeing Coordinators
☐ Clinical Child Psychologists
☐ Family Services Workers
☐ I am not sure

Did the staff member spend extra time with you talking about or working through the challenges?

☐ Yes ☐ No ☐ I don't remember

Did the staff member connect you to a different service or organisation for support with these challenges?

☐ Yes ☐ No ☐ I don't remember

If yes:

Where did the staff member connect you to for support with these challenges? (select all that apply)

☐ Paediatricians
☐ GPs
☐ Maternal Child Health Nurses
☐ Other Nurses
☐ Lawyers
☐ Speech Pathologists
☐ Family Violence Workers
☐ Wellbeing Coordinators
☐ Clinical Child Psychologists
☐ Family Services Workers
☐ Someone outside of IPC Health, please specify __________
☐ Other, not listed here, please specify __________
☐ I am not sure

Did you go to the different service or organization?

☐ Yes, I've been
☐ Yes, I've been to some but not others
☐ No, I don't plan to go
☐ No, I'm still on a waiting list
☐ I don't remember

LIST 2: INSIDE OF THE FAMILY

Over the past 6 months, how many of the following *eight* challenges have you felt concerned about affecting your child/ren or family:

- My own physical health or disability or that of another family member inside or outside of my home
- My own challenging feelings like feeling emotional, depressed, angry, anxious, exhausted or even having strange thoughts such as harming myself or others
- The way that I (or my partner) manage my child/ren's daily routines, physical needs and their behaviour
- My child/ren being left to look after themselves too much, not having their needs met, or being given too much responsibility for their age
- Someone in my family drinking alcohol or using drugs
- Conflict or tension between members of my family
- My child/ren might be seeing or exposed to behaviour within the family/at home that frightens them like threats, bullying, yelling, screaming, putting people down, hitting, slapping, kicking, or punching
- Someone in the family having problems finding or keeping a job, insecure employment or a job that is not family friendly

☐ 1 ☐ 2 ☐ 3 ☐ 4 ☐ 5 ☐ 6 ☐ 7 ☐ 8

Thinking about the *eight* challenges we just spoke about, regardless of whether or not you have been concerned about them:

In the past 6 months, has any staff member at IPC Health asked you about any of these challenges?

Yes ☐ No ☐ I don't remember ☐

If yes:

Which staff member(s)? (select all that apply)

☐ Paediatricians
☐ GPs
☐ Maternal Child Health Nurses
☐ Other Nurses
☐ Lawyers
☐ Speech Pathologists
☐ Family Violence Workers
☐ Wellbeing Coordinators
☐ Clinical Child Psychologists
☐ Family Services Workers
☐ I am not sure

Did the staff member spend extra time with you talking about or working through the challenges?

☐ Yes ☐ No ☐ I don't remember

Did the staff member connect you to a different service or organisation for support with these challenges?

☐ Yes ☐ No ☐ I don't remember

If yes:

Where did the staff member connect you to for support with these challenges? (select all that apply)

☐ Paediatricians
☐ GPs
☐ Maternal Child Health Nurses
☐ Other Nurses
☐ Lawyers
☐ Speech Pathologists
☐ Family Violence Workers
☐ Wellbeing Coordinators
☐ Clinical Child Psychologists
☐ Family Services Workers
☐ Someone outside of IPC Health, please specify ___________
☐ Other, not listed here, please specify ___________
☐ I am not sure

Did you go to the different service or organization?

☐ Yes, I've been
☐ Yes, I've been to some but not others
☐ No, I don't plan to go
☐ No, I'm still on a waiting list
☐ I don't remember

<u>LIST THREE: BROADER SOCIAL NEEDS</u>

Over the past 6 months, how many of the following *<u>three</u>* challenges have you felt concerned about affecting your child/ren or family:

- Issues with visa or immigration for someone in my family or myself.
- My own or a family member's court appearances as a defendant, being on bail, parole or spending time in prison.
- Someone in the family having problems finding or keeping a job, insecure employment or a job that is not family friendly

☐ 1 ☐ 2 ☐ 3

Thinking about the *three* challenges we just spoke about, regardless of whether or not you have been concerned about them:

In the past 6 months, has any staff member at IPC Health asked you about any of these challenges?

Yes ☐ No ☐ I don't remember ☐

If yes:

Which staff member(s)? (select all that apply)

☐ Paediatricians
☐ GPs
☐ Maternal Child Health Nurses
☐ Other Nurses
☐ Lawyers
☐ Speech Pathologists
☐ Family Violence Workers
☐ Wellbeing Coordinators
☐ Clinical Child Psychologists
☐ Family Services Workers
☐ I am not sure

Did the staff member spend extra time with you talking about or working through the challenges?

☐ Yes ☐ No ☐ I don't remember

Did the staff member connect you to a different service or organisation for support with these challenges?

☐ Yes ☐ No ☐ I don't remember

If yes:

Where did the staff member connect you to for support with these challenges? (select all that apply)

☐ Paediatricians
☐ GPs
☐ Maternal Child Health Nurses
☐ Other Nurses
☐ Lawyers
☐ Speech Pathologists
☐ Family Violence Workers
☐ Wellbeing Coordinators
☐ Clinical Child Psychologists
☐ Family Services Workers
☐ Someone outside of IPC Health, please specify __________
☐ Other, not listed here, please specify __________
☐ I am not sure

Did you go to the different service or organization?

☐ Yes, I've been
☐ Yes, I've been to some but not others
☐ No, I don't plan to go
☐ No, I'm still on a waiting list
☐ I don't remember

YOUR CHILD

What is the name and date of birth of the child aged 0-8 years old that is currently requiring the most support?

Name: ___________ Date of birth: ___________ ☐ I don't have any children, I am currently pregnant

	Excellent	Very good	Good	Fair	Poor
In general, would you say your child's health is:	☐	☐	☐	☐	☐

For each item, please mark the box for Not True, Somewhat True or Certainly True. It would help us if you answered all items as best you can even if you are not absolutely certain. Please give your answers on the basis of your child's behaviour over the last six months.

	Not true	Somewhat true	Certainly true
• Considerate of other people's feelings	☐	☐	☐
• Restless, overactive, cannot stay still for long	☐	☐	☐
• Often complains of headaches, stomach-aches or sickness	☐	☐	☐
• Shares readily with other children, for example toys, treats, pencils	☐	☐	☐
• Often loses temper	☐	☐	☐
• Rather solitary, prefers to play alone	☐	☐	☐
• Generally well behaved, usually does what adults request	☐	☐	☐
• Many worries or often seems worried	☐	☐	☐
• Helpful if someone is hurt, upset or feeling ill	☐	☐	☐
• Constantly fidgeting or squirming	☐	☐	☐
• Has at least one good friend	☐	☐	☐
• Often fights with other children or bullies them	☐	☐	☐
• Often unhappy, depressed or tearful	☐	☐	☐
• Generally liked by other children	☐	☐	☐
• Easily distracted, concentration wanders	☐	☐	☐
• Nervous or clingy in new situations, easily loses confidence	☐	☐	☐
• Kind to younger children	☐	☐	☐

Child and Family Hub

• Often argumentative with adults	☐	☐	☐
• Picked on or bullied by other children	☐	☐	☐
• Often volunteers to help others (parents, teachers, other children)	☐	☐	☐
• Can stop and think things out before acting	☐	☐	☐
• Can be spiteful to others	☐	☐	☐
• Gets along better with adults than with other children	☐	☐	☐
• Many fears, easily scared	☐	☐	☐
• Good attention span, sees chores or homework through to the end	☐	☐	☐

Overall, do you think that your child has difficulties in one or more of the following areas: emotions, concentration, behaviour or being able to get on with other people?

No	Yes – minor difficulty	Yes – definite difficulties	Yes – severe difficulties
☐	☐	☐	☐

If you have answered "Yes", please answer the following questions about these difficulties:
How long have these difficulties been present?

Less than a month	1-5 months	6-12 months	Over a year
☐	☐	☐	☐

Do the difficulties upset or distress your child?

Not at all	Only a little	Quite a lot	A great deal
☐	☐	☐	☐

Do the difficulties interfere with your child's everyday life in the following areas?

	Not at all	Only a little	Quite a lot	A great deal
• Home life	☐	☐	☐	☐
• Friendships	☐	☐	☐	☐
• Learning	☐	☐	☐	☐
• Leisure activities	☐	☐	☐	☐

Do the difficulties upset or distress your child?

Not at all	Only a little	Quite a lot	A great deal
☐	☐	☐	☐

YOU AND YOUR LIFE

These next questions are trying to measure how your life has been over the last 7 days.

In the last 7 days:	No difficulty	Slight difficulty	Some difficulty	A lot of difficulty	Unable
• How difficult was it for you to do day-to-day activities? *(for example, working, shopping, housework)*	☐	☐	☐	☐	☐
• How difficult was it for you to get around inside and outside? *(using any aids you usually use, for example, walking stick, frame or wheelchair)*	☐	☐	☐	☐	☐

	No	Mild	Moderate	Severe	Very severe
Please select one of the following to describe your experience in the last 7 days: I had _____ physical pain	☐	☐	☐	☐	☐

In the last 7 days:	Never	Only occasionally	Sometimes	Often	Most or all of the time
• I felt lonely	☐	☐	☐	☐	☐
• I felt I had no control over my day to day life *(having the choice to do things or have things done for you as you like and when you want)*	☐	☐	☐	☐	☐
• I had trouble concentrating/thinking clearly	☐	☐	☐	☐	☐
• I felt anxious	☐	☐	☐	☐	☐
• I felt exhausted	☐	☐	☐	☐	☐
• I felt sad/depressed	☐	☐	☐	☐	☐

	Excellent	Very good	Good	Fair	Poor
In general, would you say your own health is?	☐	☐	☐	☐	☐

The next set of questions ask about your experience with the Child and Family Hub@ IPC Health Wyndham Vale.

The Child and Family Hub@ IPC Health Wyndham Vale includes the following practitioners:

- GPs
- Paediatricians
- Nurses (including maternal child health nurses, GP practice nurses and other nurses)
- Child speech pathologists
- Child psychologists
- Wellbeing coordinators
- Lawyers

Thinking about all the practitioners you have seen at the Child and Family Hub@ IPC Health Wyndham Vale in the last 6 months, how often did they:	**Always**	**Often**	**Rarely**	**Sometimes**	**Never**
• Listen carefully to you?	☐	☐	☐	☐	☐
• Show respect for what you had to say?	☐	☐	☐	☐	☐
• Spend enough time with you?	☐	☐	☐	☐	☐

Would you recommend Child and Family Hub@ IPC Health Wyndham Vale to other families?

☐ Yes ☐ No

The next set of questions ask you about your relationship with your child.

If you have more than one child, please answer about the child that is currently requiring the most support.

	Never/ Almost never	Rarely	Sometimes	Often	Always/ Almost always
• How often do you express affection by hugging, kissing and holding your child?	☐	☐	☐	☐	☐
• How often do you hug or hold your child for no particular reason?	☐	☐	☐	☐	☐
• How often do you tell your child how happy he/she makes you?	☐	☐	☐	☐	☐
• How often do you have warm, close times together with your child?	☐	☐	☐	☐	☐
• How often do you enjoy listening to your child and doing things with him/her?	☐	☐	☐	☐	☐
• How often do you feel close to your child both when he/she is happy and upset	☐	☐	☐	☐	☐
• Does your child behave in a manner different from the way you want him/her to?	☐	☐	☐	☐	☐
• Do you think that your child's behaviour is more than you can handle?	☐	☐	☐	☐	☐
• Do you feel that you are good at getting your child to do what you want him/her to do?	☐	☐	☐	☐	☐
• Do you feel that you are in control and on top of things when you are caring for your child?	☐	☐	☐	☐	☐
• I have been angry with my child?	☐	☐	☐	☐	☐
• I have raised my voice with or shouted at my child?	☐	☐	☐	☐	☐
• When my child cries, he/she gets on my nerves?	☐	☐	☐	☐	☐
• I have lost my temper with my child?	☐	☐	☐	☐	☐
• I have left my child alone in his/her bedroom when he/she was particularly upset?	☐	☐	☐	☐	☐

Hall T, et al. BMJ Open 2022; 12:e055431. doi: 10.1136/bmjopen-2021-055431

During the past 30 day, about how often did you feel…	All of the time	Most of the time	Some of the time	A little of the time	None of the time
• …nervous?	☐	☐	☐	☐	☐
• …hopeless?	☐	☐	☐	☐	☐
• …restless or fidgety?	☐	☐	☐	☐	☐
• …so depressed that nothing could cheer you up?	☐	☐	☐	☐	☐
• …that everything was an effort?	☐	☐	☐	☐	☐
• …worthless?	☐	☐	☐	☐	☐

	No satisfaction at all — Completely Satisfied<br>1 2 3 4 5 6 7 8 9 10
• How satisfied are you with your standard of living?	☐--☐--☐--☐--☐--☐--☐--☐--☐--☐
• How satisfied are you with your health?	☐--☐--☐--☐--☐--☐--☐--☐--☐--☐
• How satisfied are you with what you are achieving in life?	☐--☐--☐--☐--☐--☐--☐--☐--☐--☐
• How satisfied are you with your personal relationships?	☐--☐--☐--☐--☐--☐--☐--☐--☐--☐
• How satisfied are you with how safe you feel?	☐--☐--☐--☐--☐--☐--☐--☐--☐--☐
• How satisfied are you with feeling part of your community?	
• How satisfied are you with your future security?	☐--☐--☐--☐--☐--☐--☐--☐--☐--☐
• How satisfied are you with your spirituality or religion?	☐--☐--☐--☐--☐--☐--☐--☐--☐--☐
	☐--☐--☐--☐--☐--☐--☐--☐--☐--☐

SERVICE USAGE IN THE PAST 6 MONTHS

This next set of questions is about different services you may have used.

In the past 6 months, have you been to any of the services below to help with your child's health or wellbeing, or any of the family life challenges we have talked about today?

☐ No ☐ Yes. If yes, please tell us the number of times you went to each service and the cost to you.

	Number of times in the past 6 months	What is the cost to your family to use this service each visit? (amount you pay over and above what you get back from Medicare and private health insurance)
GP	____visits	$______
Paediatrician	____visits	$______
Maternal and Child Health Nurse	____visits	$______
School nurse	____visits	$______
Psychologist	____visits	$______
Social worker	____visits	$______
Legal support or lawyer	____visits	$______
Support service (e.g. Family violence support, alcohol and drug support, housing support or parenting support)	____visits	$______
Phone helpline (please specify):	____calls	
Other (please specify):	____	$______

Have you or your partner (if applicable) had to take any time off from usual activities (such as paid work, study, voluntary work, housekeeping) during the last 6 months because of your child's health or well-being? Please fill in below.

	Had time off paid work?	Had time off other usual activities?
Yourself	☐ No<br>☐ Yes, ____ days off<br>☐ Not applicable	☐ No<br>☐ Yes, ____ days off<br>☐ Not applicable
Your partner	☐ No<br>☐ Yes, ____ days off<br>☐ Not applicable	☐ No<br>☐ Yes, ____ days off<br>☐ Not applicable

Thank you for your time completing this survey today. Your answers will be helpful to allow us to see if the Hub is working to better support families!

😊
