# Supplementary material for: Integrated Child and Family Hub models for detecting and responding to family adversity: protocol for a mixed-methods evaluation in two sites
Source: BMJ Open. 2022 May 19;12(5):e055431. doi: 10.1136/bmjopen-2021-055431 (PMC9125738; doi:10.1136/bmjopen-2021-055431)
Supplement: Author's manuscript [file bmjopen-2021-055431.draft_revisions.pdf]

BMJ Open

BMJ Open is committed to open peer review. As part of this commitment we make the peer review history of every article we publish publicly available.

When an article is published we post the peer reviewers' comments and the authors' responses online. We also post the versions of the paper that were used during peer review. These are the versions that the peer review comments apply to.

The versions of the paper that follow are the versions that were submitted during the peer review process. They are not the versions of record or the final published versions. They should not be cited or distributed as the published version of this manuscript.

BMJ Open is an open access journal and the full, final, typeset and author-corrected version of record of the manuscript is available on our site with no access controls, subscription charges or pay-per-view fees (http://bmjopen.bmj.com).

If you have any questions on BMJ Open's open peer review process please email info.bmjopen@bmj.com

BMJ Open

Integrated Child and Family Hub models for detecting and responding to family adversity: protocol for a mixed-methods evaluation in two sites.

Journal:	*BMJ Open*
Manuscript ID	bmjopen-2021-055431
Article Type:	Protocol
Date Submitted by the Author:	12-Jul-2021
Complete List of Authors:	Hall, Tess; Murdoch Childrens Research Institute,<br>Goldfeld, Sharon<br>Loftus, Hayley; Murdoch Children's Research Institute, Health Services; Royal Children's Hospital, Health Services<br>Honisett, Suzy<br>Liu, Hueiming; The George Institute for Global Health,<br>De Souza, Denise ; Torrens University Australia - Fitzroy Campus<br>Bailey, Cate; The University of Melbourne School of Population and Global Health<br>Reupert, Andrea; Monash University, Education<br>Yap, Marie; Monash University,<br>Eapen, Valsamma; South Western Sydney Local Health District, ICAMHS; University of New South Wales, School of Psychiatry<br>Haslam, Ric; The Royal Children's Hospital Melbourne<br>Sanci, Lena; University of Melbourne, Department of General Practice<br>Fisher, Jane; Monash University, Jean Hailes Research Unit, School of Public Health and Preventative Medicine<br>Eastwood, John; The University of Sydney,<br>Mukumbang, Ferdinand; University of the Western Cape, School of Public Health; Ferdinand C. Mukumbang,<br>Loveday, Sarah; Murdoch Childrens Research Institute<br>Jones, Renee; Murdoch Childrens Research Institute<br>Constable, Leanne; Murdoch Childrens Research Institute<br>Forell, Suzie; Health Justice Australia<br>Morris, Zoe; Monash University<br>Montgomery, Alicia; Marrickville Health Centre<br>Pringle, Glenn; IPC Health<br>Dalziel, Kim; The University of Melbourne, School of Population and Global Health<br>Hiscock, Harriet; The Royal Children's Hospital Melbourne, Centre for Community Child Health
Keywords:	Protocols & guidelines < HEALTH SERVICES ADMINISTRATION & MANAGEMENT, MENTAL HEALTH, Community child health < PAEDIATRICS, PRIMARY CARE, Child & adolescent psychiatry < PSYCHIATRY, PUBLIC HEALTH

SCHOLARONE™
Manuscripts

Integrated Child and Family Hub models for detecting and responding to family adversity: protocol for a mixed-methods evaluation in two sites.

Correspondence to Professor Harriet Hiscock

Murdoch Children's Research Institute: 50 Flemington Rd, Parkville, Victoria, Australia 3052

harriet.hiscock@rch.org.au

Word count: 4000

Author name	Department	Organisation	City, State, Country
Teresa Hall	Centre for Community Child Health	Murdoch Children's Research Institute	Parkville, Victoria, Australia
Sharon Goldfeld	Centre for Community Child Health	Murdoch Children's Research Institute	Parkville, Victoria, Australia
Hayley Loftus	Centre for Community Child Health	Murdoch Children's Research Institute	Parkville, Victoria, Australia
Suzy Honisett	Centre for Community Child Health	Murdoch Children's Research Institute	Parkville, Victoria, Australia
Huei Ming Liu	Sydney Institute for Women, Children and their families[a] ;The George Institute for Global Health[b]	Sydney Local Health District[a]; University of New South Wales[b]	Marrickville, New South Wales, Australia
Denise De Souza	Centre for Research in Education and Sustainability	Torrens University	Melbourne City, Victoria, Australia
Cate Bailey	Melbourne School of Population and Global Health	University of Melbourne	Parkville, Victoria, Australia
Andrea Reupert	Krongold Clinic	Monash University	Notting Hill, Victoria, Australia
Marie Yap	School of Psychological Sciences	Monash University	Clayton, Victoria, Australia
Valsamma Eapen	Infant Child and Adolescent Psychiatry	University of New South Wales	Sydney, New South Wales, Australia
Ric Haslam	Department of Paediatrics	University of Melbourne	Parkville, Victoria, Australia
Lena Sanci	Department of General Practice	University of Melbourne	Parkville, Victoria, Australia
Jane Fisher	Global Health and Women's Health	Monash University	Melbourne, Victoria, Australia

For peer review only - http://bmjopen.bmj.com/site/about/guidelines.xhtml

John Eastwood	Clinical Services Integration and Population Health	Sydney Local Health District	Camperdown, New South Wales, Australia
Ferdinand C Mukumbang	Department of Global Health	University of Washington	Seattle, USA
Sarah Loveday	Centre for Community Child Health	Murdoch Children's Research Institute	Parkville, Victoria, Australia
Renee Jones	Centre for Community Child Health	Murdoch Children's Research Institute	Parkville, Victoria, Australia
Leanne Constable	Centre for Community Child Health	Murdoch Children's Research Institute	Parkville, Victoria, Australia
Suzie Forell	[no department for [a]]; School of Law[b]	Health Justice Australia[a]; University of New South Wales[b]	Darlinghurst, Sydney, Australia
Zoe Morris	Faculty of Education	Monash University	Clayton, Victoria, Australia
Alicia Montgomery	Marrickville Health Centre	Sydney Local Health District	Marrickville, New South Wales, Australia
Glenn Pringle	Innovation and Community Care	IPC Health	Wyndham Vale, Victoria, Australia
Kim Dalziel	Melbourne School of Population and Global Health	University of Melbourne	Parkville, Victoria, Australia
Harriet Hiscock	Centre for Community Child Health	Murdoch Children's Research Institute	Parkville, Victoria, Australia

Abstract

Introduction: Integrated community health care Hubs may offer a 'one stop shop' for service users with complex health and social needs, and more efficiently use service resources. Various policy imperatives exist to implement Hub models of care, however, there is a dearth of research specifically evaluating Hubs targeted at families experiencing adversity. To contribute to building this evidence, we propose to co-design, test and evaluate integrated Hub models of care in two Australian community health care services in low socioeconomic areas that serve families experiencing adversity: Wyndham Vale in Victoria and Marrickville in NSW.

Methods and analysis: This multi-site convergent mixed-methods study will run over three research phases, guided by an interdisciplinary conceptual framework. The study utilises caregiver and Hub practitioner surveys at baseline, and then 6- and 12-months after Hub implementation and in-depth interviews at 12-months. Two stakeholder groups will be recruited: caregivers (n = 100-200 per site) and Hub practitioners (n = 20-30 per site). The

For peer review only - http://bmjopen.bmj.com/site/about/guidelines.xhtml

intervention is a co-located Hub providing health, social, legal and community services. There is no comparator. The primary outcomes are caregiver-reported: (i) identification of, (ii) interventions received and/or (iii) referrals received for adversity from Hub practitioners. The study also assesses child, caregiver, practitioner, and system outcomes. Primary and secondary outcomes will be assessed by examining change in proportions/means from baseline to 6-months, from 6- to 12-months, and from baseline to 12-months. Service linkages will be analysed using social network analysis. The costs of Hub implementation and a health economics analysis of unmet need will be conducted. Thematic analysis will be employed to analyse qualitative data.

Ethics and dissemination: Royal Children's Hospital and Sydney Local Health District ethics committees have approved the study (HREC/ 62866/RCHM-2020). Participants and stakeholders will receive results through meetings, presentations and publications.

Trial registration number: ISRCTN55495932

Strengths and weaknesses of this study

- The 'Child and Family Hub' study is the first to our knowledge to co-design, test and evaluate two integrated Hub models of care for improving the detection of and response to family adversity for children aged 0-8 years.
- Implementation and evaluation in two community health services across two Australian states will maximise the generalisability of findings for different community and service contexts.
- The interdisciplinary conceptual framework underpinning this study strengthens its empirical robustness through synthesis of approaches from many disciplines to capture the complexity of the Hub models.
- The study has no comparison group which means that any observed changes for primary and secondary outcomes cannot be directly attributed to the Hub models of care.
- The English-language inclusion criterion for participants limits our ability to engage culturally and linguistically diverse caregiver and practitioners.

For peer review only - http://bmjopen.bmj.com/site/about/guidelines.xhtml

Introduction

Family adversity and its impact on mental health

Family adversity includes a range of adverse childhood experiences (ACEs) such as child maltreatment (e.g., physical, verbal or sexual abuse), household dysfunction (e.g., parental mental illness, family substance abuse), community dysfunction (e.g., witnessing physical violence, discrimination), peer dysfunction (e.g., stealing, bullying) and socio-economic deprivation.[1] Family adversity impacts both child physical and mental health, increasing the risk of anxiety, depression and suicidality in childhood and across the life course. [2-7] Exposure to multiple ACEs predicts greater odds of poorer mental versus physical health outcomes in adult life.[8] Experiences of family adversity also negatively affect child neurological and physiological development and educational outcomes,[9] leading to poor life-long health and mental health.[10] Interventions aimed at reducing or preventing the impact of adversity on children could avert a substantial proportion of the population burden of mental illness[5], and build on evidence demonstrating the health and economic benefits of investing early in life.[11]

Detection and response to family adversity

As part of an Australian Centre of Research Excellence (CRE) in Childhood Adversity and Mental Health, we will develop, test and evaluate an approach to improving the detection of and response to adversity in children aged 0-8 years and their families. Effective detection of adversity is crucial to the provision of appropriate, timely and evidence-based responses to the effects of adversity. However, major service barriers exist to the detection of, and responses to, adversity, including service time pressures, lack of practitioner training, and confidence in how to discuss adversities, and practitioners' concerns about causing harm.[4 12-14] Family-level barriers also inhibit service access, including cognitive (i.e., knowledge, awareness of services, etc), psychological (i.e., fear, shame and distrust of services), financial, and structural barriers (i.e., service availability, lengthy wait times, transport, etc).[15-17] These service access barriers are amplified for families experiencing multiple adversities who require supports from health, social, education and community service systems. These systems are often siloed, which is likely to contribute to the low utilisation of services by these families.[18 19] Hence, effective detection of, and responses to adversity require intervention across multiple strata of intersectoral service systems.

For peer review only - http://bmjopen.bmj.com/site/about/guidelines.xhtml

Integrated care for families experiencing adversity

Integrated care has the potential to overcome service fragmentation and respond to holistic health and social needs for families and their children experiencing adversity. Integration is defined as care that: "*connect[s] the healthcare system (acute, primary medical and skilled) with other human service systems (e.g., long-term care, education and vocational and housing services) to improve outcomes (clinical, satisfaction and efficiency)".*[20] Integrated care initiatives can increase uptake and ongoing engagement with child health services, [21 22] improve child mental health[23 24] and offer a cost-effective and acceptable service response for families.[25 26] Integrated community health care Hubs have recently gained traction globally, driven by their appeal to offer a 'one stop shop' for service users and to efficiently use resources.[27] While there is no single definition of a Hub, the term is often used to describe a centralised service that offers a range of co-located, integrated services from multiple sectors, with linkages to external, community-based supports.[27 28] Emerging evidence exists for outcomes of Hub models of care. For example, the Healthy Homes and Neighbourhoods (HHAN) initiative in Sydney, New South Wales (NSW), is a cross-agency care coordination network with a centralised Hub nested within a broader place-based initiative and has been shown to increase service access for families experiencing adversity.[29 30]

Various policy imperatives have been established to implement and evaluate Hub models of care for child and family mental health in Australia. These include the 2021 Royal Commission into Victoria's Mental Health System (recommending implementation and evaluation of "*infant, child and family health and wellbeing multidisciplinary community-based hubs*"[31]) and the 2021 National Children's Mental Health and Wellbeing Strategy.[32] The increased rates of child mental health problems resulting from the COVID-19 pandemic underscore the need for accessible, effective mental health support in communities.[33] However, there is a dearth of research specifically evaluating Hub models of integrated care for families experiencing adversity with a particular focus on child mental health. Extant research also does not specify when and how integration of care occurs within Hubs across the layers of a service ecological system (i.e., clinical, professional, organisational and systems integration[31]), and which types of integration in co-located Hub models drive observed changes for children and families.

For peer review only - http://bmjopen.bmj.com/site/about/guidelines.xhtml

Current study

To contribute to the evidence, we propose to co-design, test and evaluate integrated Hub models of care in two Australian community health care services in low socioeconomic areas that serve families experiencing adversity, i.e., Wyndham Vale in Victoria and Marrickville in NSW. We define a Hub as a co-located service providing an intersectoral health, social, legal and community response to family adversity. The Wyndham Vale site is a Hub in a local government area and the Marrickville site is a Hub nested within a broader place-based initiative. The evidence generated from this study is crucial for increasing the conceptual precision around integrated Hub models of care and in addressing the evidence gap on their effectiveness for detecting and responding to family adversity, with possible flow-on benefits to child and caregiver mental health.

Study aims and objectives

Aim

1. To co-design, test and evaluate integrated Child and Family Hub models for detecting and responding to family adversity in children aged 0-8 years and their families in Wyndham Vale, Victoria, and Marrickville, NSW, and
2. To formulate a realist-informed program theory of how, why, for whom and under what conditions the Hubs work to detect and respond to family adversity.

Objectives

1. To assess the impact of the Hub models on:

   Primary outcomes

   1.1. caregiver-reported (i) identification of, (ii) interventions received and/or (iii) referrals received for adversity from Hub practitioners,

   Secondary outcomes

   1.2. Hub practitioner-reported (i) identification of, (ii) interventions delivered and/or (iii) referrals provided for adversity,

   1.3. caregiver-reported uptake of referrals,

   1.4. child and caregiver mental health,

   1.5. infant temperament,

   1.6. child and caregiver global health,

   1.7. caregiver parenting,

   1.8. caregiver quality of life,

For peer review only - http://bmjopen.bmj.com/site/about/guidelines.xhtml

1.9. caregiver personal wellbeing outcomes,

1.10. caregiver experience of care,

1.11. practitioner experience including confidence and competence in detecting and responding to adversity,

1.12. linkages between intersectoral Hub practitioners, and

2. To determine the costs of implementing the Hub models, and
3. To determine the extent that the Hub models are providing the intended service option (i.e., acceptability, feasibility, and fidelity to the initial Hub program theory).

Reporting in this protocol is based on the Strengthening the Reporting of Observational Studies in Epidemiology (STROBE) Statement.[34]

For peer review only

Methods and analysis

This study is a multi-site implementation and evaluation project that employs a convergent mixed-methods design conducted over three research phases. Quantitative and qualitative data will be collected in parallel and integrated during data analysis and interpretation.[35]

Interdisciplinary conceptual framework

The study is underpinned by an interdisciplinary conceptual framework drawn from implementation science, critical realism, improvement science and collaborative co-design paradigms.[36-43] Interdisciplinary research strengthens empirical robustness through synthesis of multiple approaches and can increase the uptake of research into policy and practice.[44] The approach and rationale for the interdisciplinary framework in order of importance for this study are:

- *Implementation science*: Given the focus of our inquiry on complex Hub interventions, implementation science frameworks form the basis of our process and outcomes evaluation, including our framing and synthesis of context using the Consolidated Framework for Implementation Research (CFIR)[41].
- *Critical realism and realist evaluation*: We draw upon elements of critical realism and realist evaluation, a form of theory-driven evaluation, to theorise and examine 'what works, for whom, under what circumstances, and how'.[45-48] Realist evaluation assumes that interactions between context and intervention mechanisms produce the outcomes of an intervention.[46 47 49 50] We focus our inquiry on the key context-

For peer review only - http://bmjopen.bmj.com/site/about/guidelines.xhtml

intervention-mechanism-outcome (CIMO) configurations discussed below. We complement realist evaluation methods with critical realism[51 52] to understand whether historical aspects of each locality influence the design and workings of each Hub. This overcomes a key shortcoming of realist evaluation, that is, under theorising the multiple levels of social reality surrounding each Hub.[53 54]

- *Improvement science*: We use improvement science methods to implement agile learning and testing cycles that will iterate the Hub models and embed learning systems into practice.[42]
- *Collaborative co-design:* We use these principles and methods to co-design the Hub models and promote stakeholder engagement throughout the research process.

Figure 1 displays the three study phases based on Pawson and Tilley's [45] realist evaluation framework: (1) Development of the initial Hub program theory through formative research; (2) Testing of the initial Hub theory using empirical data; and (3) Refinement of the initial Hub theory. Using realist evaluation as a guide to the phases of research and nesting other evaluation approaches within this - i.e. critical realism in phase 1, formative research and improvement science in phase 2, and implementation science through each phase - provides the interdisciplinary conceptual framework to the overall design.

For peer review only - http://bmjopen.bmj.com/site/about/guidelines.xhtml

Phase 1: Develop initial Hub theory (Oct 2020 – Aug 2021)

- Historical and current state needs assessment through online group discussions, interviews and meetings with intersectoral practitioners and caregivers
- Review and selection of focus CIMO configurations

Phase 2: Test initial Hub theory (Aug 2021 – Dec 2023)

Design: Convergent mixed-methods

Implementation, Context and Mechanism – data sources

- Caregiver baseline survey
- Hub practitioner baseline survey
- Process evaluation through routine data sources, observation, and Plan Do Study Act (PDSA) cycles
- In-depth interviews with Hub practitioners and caregivers

Outcomes

- Caregiver outcomes survey
- Hub practitioner outcomes survey
- In-depth interviews with Hub practitioners and caregivers

Phase 3: Refine Hub model through local knowledge translation (Dec 2022-Dec 2023)

- Validate Hub theory through ongoing knowledge translation activities (e.g., forums, workshops, webinars)

Figure 1 Evaluation processes and phases

Phase 1: Development of initial Hub program theory through formative research

The initial Hub program theory was developed through an intensive formative research phase, collecting contextual information and co-designing the Hub models with intersectoral (e.g., health, social, legal and education) practitioners and caregivers from each local community. This occurred from October 2020 to June 2021 in Victoria and January to August 2021 in NSW. First, a historical and current needs assessment of the health, social, legal and community contexts of each site was conducted through online group discussions, individual interviews and meetings with key stakeholders including intersectoral practitioners and caregivers. We used CFIR[41] to capture and synthesise these data into barriers and

For peer review only - http://bmjopen.bmj.com/site/about/guidelines.xhtml

enablers across the outer setting, inner setting, individual level, process and intervention components.

Second, we reviewed the HHAN program theories and other relevant realist literature focused on integrated care for children and families experiencing adversity[23 30 55-58] to identify key CIMO configurations relevant to the Hubs. For pragmatic reasons, we focus our process and outcome evaluation on three key mechanisms and associated CIMO configurations in the initial program theory. Namely, (1) trust between caregivers and the service/practitioners, and between practitioners, (2) knowledge acquisition and motivation of practitioners, and (3) perceived benefits of collaboration by practitioners. Figure 2 displays the initial realist-informed program theory. We then formulated the logic model displayed in Figure 3 proposing how the Hub models will be investigated and are theorised to contribute to outcomes. The logic model includes how immediate outcomes, i.e., increased detection and response to adversity are hypothesized to lead to our intended outcomes, including increased uptake of services and improved child and caregiver mental health and caregiver quality of life.

Third, Hub models were then developed and refined through a collaborative co-design process with intersectoral practitioners, caregivers and children at each site. The co-design process focused on the client journey through each proposed Hub and the workforce capacities and infrastructure needed to implement the Hub. In Wyndham Vale, the Hub model was co-designed through an intensive 10-week series of co-design workshops and consultations with local caregivers and practitioners using human-centred design processes.[36-38] In Marrickville, the Hub model will be co-designed through focus group discussions and workshops employing the Nominal Group Technique consensus method [59] to prioritise and develop implementation strategies.

The theorised Hub models comprise:

- *Family and community partnerships*: intentional creation and strengthening of connections between the Hub and caregivers, community groups and individuals. These partnerships also involve Hub practitioners working in partnership with families during clinical practice.

For peer review only - http://bmjopen.bmj.com/site/about/guidelines.xhtml

- *Entry to the Hub:* a 'no wrong door' approach in which caregivers are safely engaged in a conversation about adversity and provided with support and/or referrals regardless of how they enter the Hub.
- *Workforce development*: capacity building and training of Hub practitioners to engage families in a safe and respectful conversation to identify adversities and connect families to relevant support.
- *Case-based discussions:* monthly professional development with intersectoral Hub practitioners to embed training learnings into practice and facilitate between-practitioner referrals (i.e., 'warm referrals').
- *Referral pathways into and out of the Hub*: systematic mapping of available health, community and social sector supports in the local area, linked to training of Hub practitioners to use this information in their practice.
- *Care navigation*: In Wyndham Vale, a Wellbeing Coordinator will support caregivers to identify the holistic needs of their child and/or family and assist them to navigate relevant supports in the community, social and health sectors. In Marrickville, care navigation may include a Wellbeing Coordinator or a caregiver virtual application (depending on resources) that provides a risk stratification and recommendations of relevant services.

For peer review only

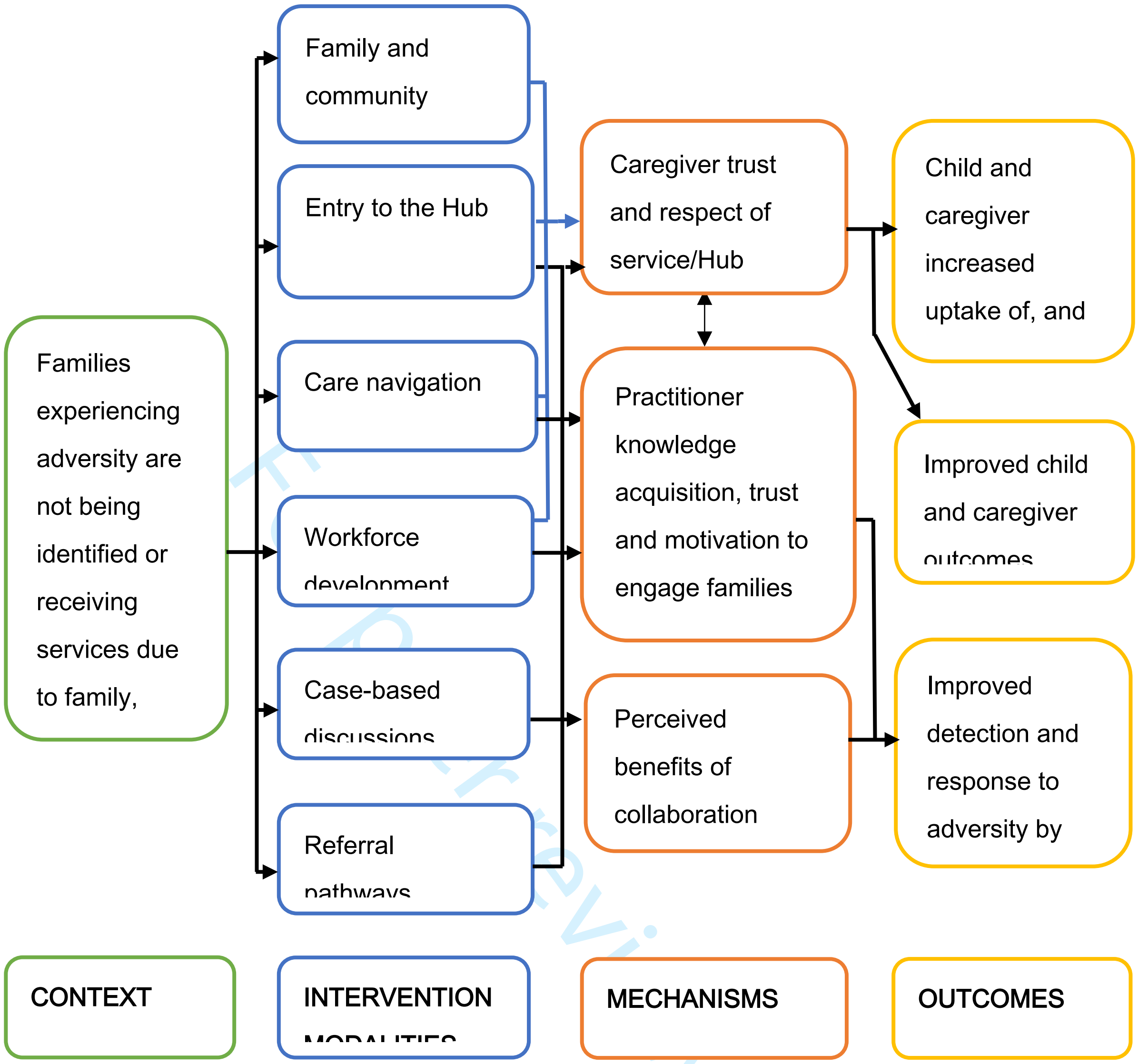

Figure 2. Initial Hub program theory from a realist perspective

For peer review only - http://bmjopen.bmj.com/site/about/guidelines.xhtml

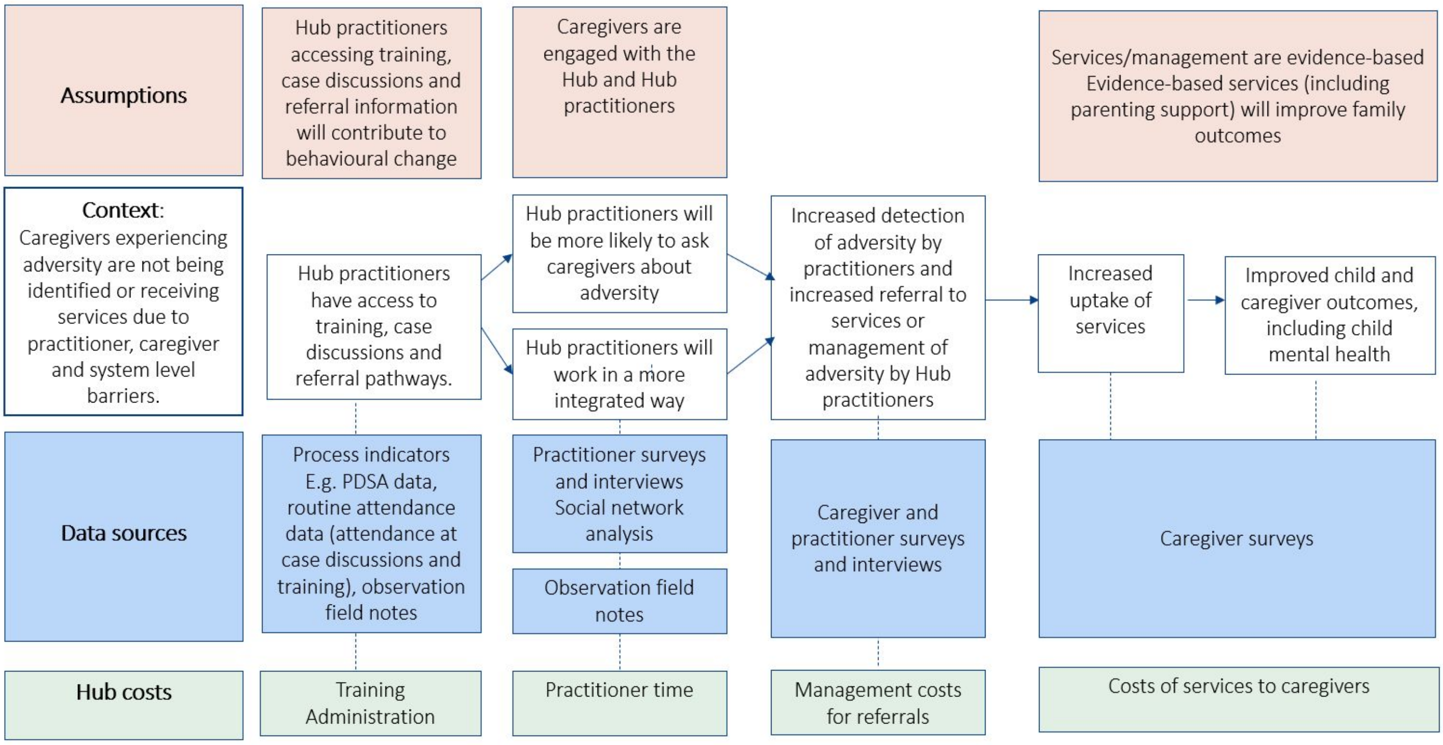

Figure 3. Logic model proposing how the Hub models will be investigated.

Phase 2: Testing of initial Hub theory using empirical data

We will test the initial program theory for the Hubs through a mixed-methods process with 6- and 12-months outcomes evaluation. The evaluation aims to assess for whom, how and why the Hub models had an impact (if any) in the two different contexts of Wyndham Vale and Marrickville across the child, caregiver, practitioner, and system level outcomes specified in Figures 2 and 3, and Table 1.

Outcome evaluation

Design

The study is a mixed-methods repeated measures evaluation that utilises caregiver and Hub practitioner surveys at baseline, 6- and 12-months after Hub implementation begins and in-depth interviews at 12-months.

Participants

We aim to recruit two stakeholder groups: caregivers (*n* = 100-200 per site) and Hub practitioners (*n* = 20-30 per site).

For peer review only - http://bmjopen.bmj.com/site/about/guidelines.xhtml

Caregivers

Potential caregiver participants are those who: (i) care for a child aged 0-8 years, including pregnant women, (ii) access universal or specialist services provided in the Hub, such as General Practitioners (GPs), paediatricians, lawyers, etc, and (iii) can understand written or spoken English-language. The English-language criterion is a limitation of our study; however, funding is not available to provide interpreters. Further, culturally and linguistically diverse persons participated in an informed and meaningful way in phone-based or in-person surveys for HHAN (personal communication Prof Eastwood).

Given the preventative focus of this study, caregivers will not be screened for adverse experiences as an inclusion criterion. Instead, caregivers will report the frequency of fifteen adversity types in the baseline survey. These adversity types are derived from the Parent Engagement Resource[60] and include challenges with: social support, finances, housing, employment, family physical health, parent mental health, parenting, child neglect, alcohol and substance use, family relationships, family violence, child abuse, visa and immigration issues, crime issues and discrimination.

Hub practitioners

Potential practitioner participants are those who: (i) work in any of the intersectoral services provided in each Hub, including GPs, nurses, paediatricians, allied health professionals, social workers, lawyers, and (ii) can understand written or spoken English-language.

Sample size calculation

As this study is testing the Hub models using a non-experimental design, we have not conducted a formal sample size calculation. However, the sample size of 200-400 caregivers is likely to provide rich data on primary and secondary outcomes.

Recruitment and consent

The study is designed to accommodate a range of participants, many of whom may have complex life circumstances.[61] Figure 4 presents the caregiver participant study flow. We will recruit caregivers from the waiting rooms of each Hub and through Hub practitioners who will ask their clients' permission for the research team to contact them. Researchers will approach potential caregiver participants (in the waiting room or on the telephone), show and/or email them an informational video and study information pack (including a written Caregiver Participant Information Consent Form, PICF), and invite them to take part in the

For peer review only - http://bmjopen.bmj.com/site/about/guidelines.xhtml

study. Participants can provide informed consent verbally on the phone or in-person, or in writing by clicking through the online consent form preceding the survey.

We will invite practitioners working in each Hub who have been identified and recruited by the study team or health centre managers. We will send an invitation email to potential practitioner participants with the Practitioner PICF attached before they attend the workforce training. Practitioner participants will provide written (online or hard copy) informed consent prior to completing the baseline survey.

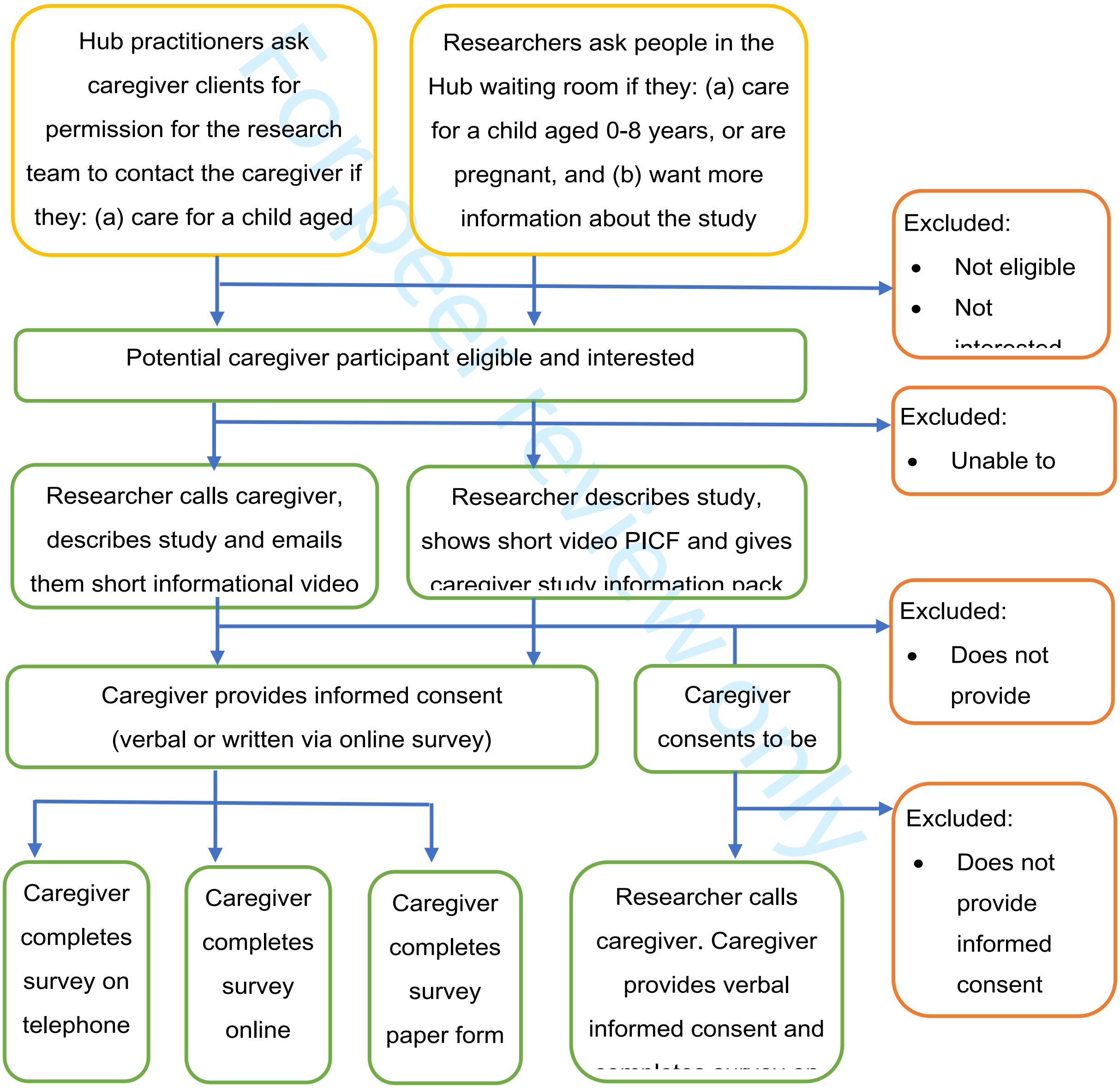

Figure 4. Caregiver participant study flow

For peer review only - http://bmjopen.bmj.com/site/about/guidelines.xhtml

Outcome assessment

The study will assess child, caregiver, practitioner, and systems outcomes summarised in Table 1.

Data collection

Caregiver and Hub practitioner surveys

The Caregiver and Hub practitioner baseline and outcome surveys consisting of the measures outlined in Table 1 will be built in REDCap[62] hosted by Murdoch Children's Research Institute. After completing an informed consent process, caregivers can complete the baseline survey: in paper form, over the telephone with a researcher or online. Practitioners can complete their baseline survey in paper form or online via REDCap. The practitioner survey will be sent towards the end of baseline data collection to minimise the potential impact of the survey on clinical practice prior to the Hub testing period. Caregiver and practitioner participants will be contacted by telephone, email or text message by the research team 6- and 12-months after the Hub implementation begins and invited to complete the follow up survey.

Caregiver and Hub practitioner interviews

Twelve-months after Hub implementation begins, we will conduct realist-informed semi-structured interviews with 18-24 caregivers and 5-10 Hub practitioners per site.[63] We will purposively sample participants to capture caregivers from diverse cultural backgrounds, experiences of adversities and varying levels of engagement with intersectoral services. The interviews aim to uncover (i) the mechanisms and contextual elements relevant to the Hub models (to inform theory development), (ii) acceptability and feasibility of the Hub, (iii) their experience of being asked about adversity, the support offered to them, and the process of referrals including barriers and enablers to uptake (caregivers only), and (iv) confidence and competence in detecting and supporting families experiencing adversity (practitioners only).

Table 1 Primary and secondary outcomes

PRIMARY OUTCOMES	BASELINE ASSESSMENT		6- AND 12-MONTHS POST HUB IMPLEMENTATION BEGINS	
	Caregiver survey	Practitioner survey	Caregiver survey	Practitioner survey
Caregiver				

For peer review only - http://bmjopen.bmj.com/site/about/guidelines.xhtml

**Identification of adversity**	Increase in the proportion of caregivers who report being asked by a service provider about adversity in the past six months	X		X	
**Intervention for adversity**	Increase in the proportion of caregivers who report spending extra time with or receiving an intervention from a Hub service provider for adversity in the past six months	X		X	
**Referrals for adversity**	Increase in the proportion of caregivers who report receiving a referral to an intersectoral service for adversity in the past six months	X		X	
SECONDARY OUTCOMES					
Child					
**Infant temperament**	Increase in the proportion of caregivers who report their infant is easier/much easier than average; assessed through single caregiver-reported item on infant temperament; has a moderate correlation (r=0.51) with the Easy-Difficult Scale (EDS) of Australian version short form of Revised Infant Temperament Questionnaire[64]; completed for one child aged 0-8 in each family 0 to < 2 years	X		X	
**Child mental health**	Decrease in the mean scores for caregiver-reported internalising or externalising symptoms for their child.<br>Completed for one child in each family.<br>For children aged 0 to < 2 years: Ages & Stages Questionnaire Social-Emotional Second Edition (ASQ-SE2)[65]<br>For children aged ≥ 2 to 8 years: Strengths & Difficulties Questionnaire [66]	X		X	
**Global health**	Increase in the mean scores of caregiver-reported general child health; assessed through single item	X		X	

For peer review only - http://bmjopen.bmj.com/site/about/guidelines.xhtml

	(GHQ-S1) from Child Health Questionnaire [67]; completed for one child aged 0-8 in each family				
Caregiver					
**Uptake of referrals**	Increase in the proportion of caregivers who report uptake of referrals to other services in the past six months.	X		X	
**Mental health**	Decrease in the mean scores of caregiver-reported psychological distress as assessed by Kessler Psychological Distress Scale 6 (K6); 6-item [68]	X		X	
**Global health**	Increase in means scores of caregiver-reported general health; assessed through single item of the Short Form Health Survey (SF-12)[69]	X		X	
**Parental warmth, parenting hostility and efficacy**	Increase in mean scores of parental warmth and efficacy; decrease in mean scores on parenting hostility. Three self-report subscales drawn from the Longitudinal Study of Australian Children (LSAC), Australia's first nationally representative longitudinal study of child development which will allow for national comparisons of the study data [70]; assessing parental warmth, parenting hostility and efficacy. Parental warmth (6-items), parenting hostility (5-items) and parenting efficacy (4-items)	X		X	
**Quality of life**	Increase in mean caregiver quality of life scores calculated from the EuroQol Health and Wellbeing Instrument Short Form (EQ-HWB-S) [71]	X		X	
**Caregiver experience**	Caregiver reported acceptability and feasibility of the Hub; Increase in the proportion of caregivers who report their satisfaction with Hub care as measured by 3 items from the Australia Bureau of Statistics Patient	X		X	

For peer review only - http://bmjopen.bmj.com/site/about/guidelines.xhtml

	Experiences in Australia Survey [72]				
**Personal wellbeing**	Increase in mean scores of caregiver-reported personal wellbeing outcomes measured by the Personal Wellbeing Index; 7 items [73]	X		X	
Practitioner					
**Identification of adversity**	Increase in the proportion of practitioners who report asking about adversity in the past six months		X		X
**Intervention for adversity**	Increase in the proportion of practitioners who report spending extra time or providing an intervention for adversity in the past six months		X		X
**Referrals for adversity**	Increase in the proportion of practitioners who report referring caregivers to an intersectoral service for adversity in the past six months		X		X
**Practitioner experience**	Practitioner reported acceptability and feasibility of the Hub; Increase in the proportion of practitioners who report feeling confident and competent to detect and support families experiencing adversity		X		X
System level					
**Strength and structure of intersectoral service linkages**	Increase in the number and strength of service linkages between Hub practitioners as assessed through social network analysis (SNA) indicators for network density, degree, centrality and betweenness, and map of linkages between Hub practitioners based on i) contact, (ii) referrals to, (iii) referrals from, and (iv) quality of the relationship[74-77]		X		X
**Health economics outcomes**	Costs of implementation of the Hub models, caregiver-reported intersectoral service usage and value of unmet need	X		X	

For peer review only

For peer review only - http://bmjopen.bmj.com/site/about/guidelines.xhtml

Quantitative data analysis

Statistical analyses will be conducted using STATA or R statistical software packages.[78 79] The baseline characteristics of the caregivers, children and practitioners will be described using the mean, median and interquartile range for continuous data and proportions for categorical data. Primary and secondary outcomes will be assessed by examining change in proportions/means from baseline to 6-, from 6- to 12- and from baseline to 12-months. We will also conduct linear regression analyses to assess the association between each caregiver-reported primary outcome at 6- and 12-months with the outcomes of child and caregiver mental health and caregiver quality of life, at 6 and 12-months, respectively.

Quantitative measures of the strength and structure of service linkages between Hub practitioners will be analysed using social network analysis (SNA) software package UCINET.[80] SNA is a complex systems discipline and quantitative methodology used to measure networked relationships between organisations and individual actors in health service and policy settings.[74-77] UCINET[80] will calculate indicators (i.e., density, degree, centrality and betweenness) for the network and each Hub practitioner based on: i) contact, (ii) referrals to and from, and (iii) quality of the relationship. These indicators and analytical approach are recommended for descriptive social network analysis.[74-77]

Health economics analysis

Costs of the Hubs will be identified, including workforce training, administration for referrals, personnel to oversee clinical implementation and other relevant costs. Downstream costs of services and potential cost-offsets will be collected and analysed to obtain the cost per additional child/or caregiver attending the Hub. Quality of life will be measured using the carer-specific measure (EQ-HWB-S[71]). An analysis of the value of unmet need will be conducted by determining whether (i) the child and family's needs were met, (ii) no needs, or (iii) unmet needs using the ASQ or SDQ to determine perceived need compared to the level of service use.

Qualitative data analysis

Individual interviews will be transcribed verbatim by a transcription service and imported into N-Vivo Release 1.0 [81] to assist analysis. Researchers will employ Braun and Clarke's six-stage reflexive thematic analysis [82] to analyse the qualitative interview data. In line with the

For peer review only - http://bmjopen.bmj.com/site/about/guidelines.xhtml

critical realist philosophy underpinning this study, we will employ inductive coding and deductive (*a priori*) coding based on the CIMO configurations for the Hub.

Process evaluation and quality improvement cycles

We will conduct a process evaluation using multiple data sources, including routinely collected data (e.g., attendance rates at training, visits to the Wellbeing Coordinator) and observation field notes of Hub practitioner clinical practice, the training sessions and the case-based discussions. The process evaluation examines the acceptability, feasibility, and fidelity of implementation for each Hub component shown in the initial Hub program theory in Figure 2. The process evaluation will also include realist analytical methods in which observed outcomes are explained by looking into the mechanisms and contextual elements contributing to the focus CIMOs for this study.

We will also conduct improvement cycles guided by the Model for Improvement.[42] Small improvement teams at each site will establish their specific improvement aims and measurements. Once the aims are agreed, the team will undertake short learning cycles via Plan-Do-Study-Act (PDSA) cycles.[42] By project end, learning cycles will be embedded as part of routine Hub service delivery, making the Hub more likely to be sustained and used.[40] To maximise rigor, we will triangulate data collected through the process evaluation and PDSA cycles with outcome measures collected through the 6- and 12-month surveys.[83]

Phase 3: Refinement of initial Hub program theory

We will use process and outcome evaluation data to refine the initial Hub program theory. Further testing will be undertaken by explicitly seeking to confirm or contradict theories throughout the PDSA cycles, in-depth interviews and knowledge translation activities (e.g., forums, workshops, webinars). A key strength of our study is the assessment of outcomes across multiple levels of the Hub intervention. The triangulation of multiple data sources to evaluate the implementation of the Hub model serves to maximise the study's rigour and confidence in our findings.[83 84]

Patient and public involvement

Caregivers and practitioners were actively involved in the co-design of the Hub models and are members of local advisory groups at each site which oversee the study design, recruitment, piloting of instruments, interpretation and dissemination of findings. Their

For peer review only - http://bmjopen.bmj.com/site/about/guidelines.xhtml

engagement is aimed at ensuring the research is responsive to context and to facilitate the translation of findings into practice. [85]

Ethics and dissemination

The study protocol has been approved by The Royal Children's Hospital Human Research Ethics Committee (HREC/ 62866/RCHM-2020) and Sydney Local Health District (HREC/ 62866/RCHM-2020). All participants will be asked to provide written or verbal informed consent prior to completing the surveys and interviews. Each participant will be allocated a participant identification number that is kept securely under password protection accessible only to the research team.

Confidentiality

Participant confidentiality is strictly held in trust by the participating investigators, research staff, and the sponsoring institution and their agents. The study protocol, documentation, data and all other information generated will be held in strict confidence. No information concerning the study or the data will be released to any unauthorised third party other than MCRI, without prior approval by the participant.

Data access

All investigators will be able to access cleaned study data for analysis. Data will be housed on Murdoch Children's Research Institute's secure network or via file transfer, and all datasets will be password protected. To ensure confidentiality, dispersed data files will not include identifying participant information.

Participant distress

While we do not anticipate any major risks to participants, the surveys and individual interviews may raise issues that are uncomfortable, upsetting, or frustrating for some. To reduce potential for distress to participants, the study team will provide clear explanations about why the study is being conducted, how the information will be used, and the kinds of questions that will be asked. If the participant becomes distressed at any point, the researchers will empathise with the participant, stop the survey or interview if requested, and discuss ways in which they might get support from family, friends or formal services.

For peer review only

Dissemination

We will publish study findings in peer-reviewed journals and present papers at national and international conferences. At a local level in Wyndham Vale and Marrickville, we will communicate project learnings to stakeholders via presentations, community social media, and research summaries. We will provide research summaries to local and statewide media and in social media posts. A knowledge translation strategy will disseminate findings using a range of mediums including statewide and national Hub networks and regular engagements with relevant government departments. The strategy is intended to support the scale up of effective components to other community health services in Victoria, NSW and across Australia.

Acknowledgements

We thank all families, practitioners and researchers who are working together on the Child and Family Hubs project, and members of the advisory and implementation groups at each site for their oversight and guidance. We acknowledge Professor Anthony Jorm as the Chief Investigator responsible for the first stream of work (Theme A) conducted by the CRE Childhood Adversity and Mental Health.

Full references

1. Karatekin C, Hill M. Expanding the original definition of adverse childhood experiences (ACEs). *Journal of Child & Adolescent Trauma* 2019;12(3):289-306.

2. Lawrence D, Hafekost J, Johnson SE, et al. Key findings from the second Australian child and Adolescent Survey of Mental Health and Wellbeing. *Australian & New Zealand Journal of Psychiatry* 2016;50(9):876-86.

3. McLaughlin KA. Future directions in childhood adversity and youth psychopathology. *Journal of Clinical Child & Adolescent Psychology* 2016;45(3):361-82.

4. Jorm AF, Patten SB, Brugha TS, et al. Has increased provision of treatment reduced the prevalence of common mental disorders? Review of the evidence from four countries. *World Psychiatry* 2017;16(1):90-99.

5. Jorm AF. Why hasn't the mental health of Australians improved? The need for a national prevention strategy. *Aust N Z J Psychiatry* 2014;48(9):795-801. doi: 10.1177/0004867414546387 [published Online First: 2014/08/26]

For peer review only - http://bmjopen.bmj.com/site/about/guidelines.xhtml

6. Kessler RC, Angermeyer M, Anthony JC, et al. Lifetime prevalence and age-of-onset distributions of mental disorders in the World Health Organization's World Mental Health Survey Initiative. *World psychiatry* 2007;6(3):168.

7. Sahle BW, Reavley NJ, Li W, et al. The association between adverse childhood experiences and common mental disorders and suicidality: an umbrella review of systematic reviews and meta-analyses. *Eur Child Adolesc Psychiatry* 2021 doi: 10.1007/s00787-021-01745-2 [published Online First: 2021/02/28]

8. Hughes K, Bellis MA, Hardcastle KA, et al. The effect of multiple adverse childhood experiences on health: a systematic review and meta-analysis. *Lancet Public Health* 2017;2(8):e356-e66. doi: 10.1016/s2468-2667(17)30118-4 [published Online First: 2017/12/19]

9. Shonkoff JP, Garner AS, Siegel BS, et al. The Lifelong Effects of Early Childhood Adversity and Toxic Stress. *Pediatrics* 2012;129(1):e232-e46. doi: 10.1542/peds.2011-2663

10. Duchesne S, Vitaro F, Larose S, et al. Trajectories of Anxiety During Elementary-school Years and the Prediction of High School Noncompletion. *Journal of Youth and Adolescence* 2008;37(9):1134-46. doi: 10.1007/s10964-007-9224-0

11. Moore T, Arefadib N, Deery A, et al. The first thousand days: An evidence paper - summary. Parkville, Victoria: Centre for Community Child health, Murdoch Children's Research Institute, 2017.

12. Albaek AU, Kinn LG, Milde AM. Walking Children Through a Minefield: How Professionals Experience Exploring Adverse Childhood Experiences. *Qual Health Res* 2018;28(2):231-44. doi: 10.1177/1049732317734828 [published Online First: 2017/10/20]

13. Pearce J, Murray C, Larkin W. Childhood adversity and trauma: experiences of professionals trained to routinely enquire about childhood adversity. *Heliyon* 2019;5(7):e01900-e00. doi: 10.1016/j.heliyon.2019.e01900

14. Eismann EA, Theuerling J, Maguire S, et al. Integration of the Safe Environment for Every Kid (SEEK) Model Across Primary Care Settings. *Clin Pediatr (Phila)* 2019;58(2):166-76. doi: 10.1177/0009922818809481 [published Online First: 2018/10/30]

15. George S, Daniels K, Fioratou E. A qualitative study into the perceived barriers of accessing healthcare among a vulnerable population involved with a community centre in Romania. *International Journal for Equity in Health* 2018;17(1):41. doi: 10.1186/s12939-018-0753-9

For peer review only - http://bmjopen.bmj.com/site/about/guidelines.xhtml

16. Brown A, Rice SM, Rickwood DJ, et al. Systematic review of barriers and facilitators to accessing and engaging with mental health care among at-risk young people. *Asia Pac Psychiatry* 2016;8(1):3-22. doi: 10.1111/appy.12199 [published Online First: 2015/08/05]

17. Carrillo JE, Carrillo VA, Perez HR, et al. Defining and targeting health care access barriers. *J Health Care Poor Underserved* 2011;22(2):562-75. doi: 10.1353/hpu.2011.0037 [published Online First: 2011/05/10]

18. Alcalá HE, Valdez-Dadia A, von Ehrenstein OS. Adverse childhood experiences and access and utilization of health care. *Journal of Public Health* 2017;40(4):684-92. doi: 10.1093/pubmed/fdx155

19. Koball AM, Rasmussen C, Olson-Dorff D, et al. The relationship between adverse childhood experiences, healthcare utilization, cost of care and medical comorbidities. *Child Abuse & Neglect* 2019;90:120-26. doi: https://doi.org/10.1016/j.chiabu.2019.01.021

20. Leutz WN. Five laws for integrating medical and social services: lessons from the United States and the United Kingdom. *Milbank Q* 1999;77(1):77-110, iv-v. doi: 10.1111/1468-0009.00125 [published Online First: 1999/04/10]

21. Edwards K, Rimes T, Smith R, et al. "Improving Access to Early Childhood Developmental Surveillance for Children from Culturally and Linguistically Diverse (CALD) Background". *Int J Integr Care* 2020;20(2):3. doi: 10.5334/ijic.4696 [published Online First: 2020/04/30]

22. Edwards K, Fernandez R, Rimes T, et al. Happy, healthy, ready-working with early childhood non-government organisations for developmental surveillance for vulnerable children. *Australian Journal of Advanced Nursing, The* 2020;37(4):37-46.

23. Yonek J, Lee CM, Harrison A, et al. Key Components of Effective Pediatric Integrated Mental Health Care Models: A Systematic Review. *JAMA Pediatr* 2020;174(5):487-98. doi: 10.1001/jamapediatrics.2020.0023 [published Online First: 2020/03/10]

24. Asarnow JR, Rozenman M, Wiblin J, et al. Integrated Medical-Behavioral Care Compared With Usual Primary Care for Child and Adolescent Behavioral Health: A Meta-analysis. *JAMA Pediatr* 2015;169(10):929-37. doi: 10.1001/jamapediatrics.2015.1141 [published Online First: 2015/08/11]

25. Lane WG, Dubowitz H, Frick KD, et al. Cost effectiveness of SEEK: A primary care-based child maltreatment prevention model. *Child Abuse Negl* 2021;111:104809. doi: 10.1016/j.chiabu.2020.104809 [published Online First: 2020/11/19]

For peer review only - http://bmjopen.bmj.com/site/about/guidelines.xhtml

26. Satherley R-M, Lingam R, Green J, et al. Integrated health Services for Children: a qualitative study of family perspectives. *BMC Health Services Research* 2021;21(1):167. doi: 10.1186/s12913-021-06141-9

27. Elrod JK, Fortenberry JL. The hub-and-spoke organization design: an avenue for serving patients well. *BMC Health Services Research* 2017;17(1):457. doi: 10.1186/s12913-017-2341-x

28. Malachowski C, Skopyk S, Toth K, et al. The Integrated Health Hub (IHH) Model: The Evolution of a Community Based Primary Care and Mental Health Centre. *Community Ment Health J* 2019;55(4):578-88. doi: 10.1007/s10597-018-0339-4 [published Online First: 2018/10/03]

29. Eastwood J, Barmaky S, Hansen S, et al. Refining Program Theory for a Place-Based Integrated Care Initiative in Sydney, Australia. *Int J Integr Care* 2020;20(3):13. doi: 10.5334/ijic.5422 [published Online First: 2020/10/13]

30. Eastwood JG, Dewhurst S, Hansen S, et al. Care Coordination for Vulnerable Families in the Sydney Local Health District: What Works for Whom, under What Circumstances, and Why? *Int J Integr Care* 2020;20(4):22. doi: 10.5334/ijic.5437 [published Online First: 2020/12/19]

31. State of Victoria. Royal Commission into Victoria's Mental Health System, Final Report, Summary and recommendations. Melbourne, Victoria: State of Victoria, 2021.

32. Australian Government National Mental Health Commission. National Children's Mental Health and Wellbeing Strategy (draft). Canberra: Australian Government, 2021.

33. Cheek JA, Craig SS, West A, et al. Emergency department utilisation by vulnerable paediatric populations during the COVID-19 pandemic. *Emerg Med Australas* 2020;32(5):870-71. doi: 10.1111/1742-6723.13598 [published Online First: 2020/07/25]

34. von Elm E, Altman DG, Egger M, et al. Strengthening the Reporting of Observational Studies in Epidemiology (STROBE) statement: guidelines for reporting observational studies. *Bmj* 2007;335(7624):806-8. doi: 10.1136/bmj.39335.541782.AD [published Online First: 2007/10/20]

35. Creswell JW, Plano Clark, V.L. Designing and Conducting Mixed Methods Research. Los Angeles: SAGE 2018.

36. Plattner H, Meinel C, Leifer L. Design thinking: understand–improve–apply: Springer Science & Business Media 2011.

For peer review only - http://bmjopen.bmj.com/site/about/guidelines.xhtml

37. Roberts JP, Fisher TR, Trowbridge MJ, et al. A design thinking framework for healthcare management and innovation. *Healthc (Amst)* 2016;4(1):11-4. doi: 10.1016/j.hjdsi.2015.12.002 [published Online First: 2016/03/24]
38. Mulvale A, Miatello A, Hackett C, et al. Applying experience-based co-design with vulnerable populations: Lessons from a systematic review of methods to involve patients, families and service providers in child and youth mental health service improvement. *Patient Experience Journal* 2016;3(1):117-29.
39. Reed JE, Green S, Howe C. Translating evidence in complex systems: a comparative review of implementation and improvement frameworks. *International Journal for Quality in Health Care* 2019;31(3):173-82. doi: 10.1093/intqhc/mzy158
40. Chambers DA, Glasgow RE, Stange KC. The dynamic sustainability framework: addressing the paradox of sustainment amid ongoing change. *Implementation Science* 2013;8(1):117.
41. Damschroder LJ, Aron DC, Keith RE, et al. Fostering implementation of health services research findings into practice: a consolidated framework for advancing implementation science. *Implement Sci* 2009;4:50. doi: 10.1186/1748-5908-4-50 [published Online First: 2009/08/12]
42. Langley GJ, Moen RD, Nolan KM, et al. The improvement guide: a practical approach to enhancing organizational performance: John Wiley & Sons 2009.
43. Blamey A, Mackenzie M. Theories of Change and Realistic Evaluation. *Evaluation* 2016;13(4):439-55. doi: 10.1177/1356389007082129
44. Okamura K. Interdisciplinarity revisited: evidence for research impact and dynamism. *Palgrave Communications* 2019;5(1):141. doi: 10.1057/s41599-019-0352-4
45. Pawson R, Tilley, N.,. Realistic Evaluation. London, United Kingdom: SAGE Publications 1997.
46. Wong G, Westhorp G, Manzano A, et al. RAMESES II reporting standards for realist evaluations. *BMC medicine* 2016;14(1):96.
47. Greenhalgh T, Wong G, Jagosh J, et al. Protocol—the RAMESES II study: developing guidance and reporting standards for realist evaluation. *BMJ open* 2015;5(8):e008567.
48. Nurjono M, Shrestha P, Lee A, et al. Realist evaluation of a complex integrated care programme: protocol for a mixed methods study. *BMJ Open* 2018;8(3):e017111. doi: 10.1136/bmjopen-2017-017111 [published Online First: 2018/03/04]

For peer review only - http://bmjopen.bmj.com/site/about/guidelines.xhtml

49. Marchal B, van Belle S, van Olmen J, et al. Is realist evaluation keeping its promise? A review of published empirical studies in the field of health systems research. *Evaluation* 2012;18(2):192-212. doi: 10.1177/1356389012442444
50. Porter S. Realist evaluation: an immanent critique. *Nursing Philosophy* 2015;16(4):239-51.
51. Bhaskar R. The possibility of naturalism: A philosophical critique of the contemporary human sciences. Florence KY, USA: Routledge 1998.
52. Archer MS, Archer MS. Realist social theory: The morphogenetic approach: Cambridge university press 1995.
53. Lemire S, Kwako A, Nielsen SB, et al. What is this thing called a mechanism? Findings from a review of realist evaluations. *New Directions for Evaluation* 2020;2020(167):73-86.
54. Hinds K, Dickson K. Realist synthesis: a critique and an alternative. *Journal of Critical Realism* 2021;20(1):1-17. doi: 10.1080/14767430.2020.1860425
55. Eastwood JG, De Souza DE, Shaw M, et al. Designing Initiatives for Vulnerable Families: From Theory to Design in Sydney, Australia. *Int J Integr Care* 2019;19(3):9. doi: 10.5334/ijic.3963 [published Online First: 2019/08/02]
56. Eastwood JG, Kemp LA, Garg P, et al. A Critical Realist Translational Social Epidemiology Protocol for Concretising and Contextualising a "Theory of Neighbourhood Context, Stress, Depression, and the Developmental Origins of Health and Disease (DOHaD)", Sydney Australia. *Int J Integr Care* 2019;19(3):8. doi: 10.5334/ijic.3962 [published Online First: 2019/08/02]
57. Eastwood JG, Woolfenden S, Miller E, et al. Implementation, Mechanisms of Effect and Context of an Integrated Care Intervention for Vulnerable Families in Central Sydney Australia: A Research and Evaluation Protocol. *Int J Integr Care* 2019;19(3):11. doi: 10.5334/ijic.4217 [published Online First: 2019/08/02]
58. Tyler I, Lynam J, O'Campo P, et al. It takes a village: a realist synthesis of social pediatrics program. *Int J Public Health* 2019;64(5):691-701. doi: 10.1007/s00038-018-1190-7 [published Online First: 2018/12/26]
59. Cantrill J, Sibbald B, Buetow S. The Delphi and nominal group techniques in health services research. *International Journal of pharmacy practice* 1996;4(2):67-74.
60. Moore T, Yagnik, Halloran, et al. Developing the Parent Engagement Resource: a tool for enhancing parent-professional relationships and identifying psychosocial issues in families. *Australian Journal of Child and Family Health Nursing* 2012;9:12-16.

For peer review only - http://bmjopen.bmj.com/site/about/guidelines.xhtml

61. Price A, Bryson H, Smith A, et al. Processes for engaging and retaining women who are experiencing adversity in longitudinal health services research. *BMC health services research* 2019;19(1):1-12.

62. Harris PA, Taylor, R., Thielke, R., Payne, J., Gonzalez, N., Conde, J.G., . Research electronic data capture (REDCap) - A metadata-driven methodology and workflow process for providing translational research informatics support. *J Biomed Inform* 2009;42(2):377-81.

63. Mukumbang FC, Marchal B, Van Belle S, et al. Using the realist interview approach to maintain theoretical awareness in realist studies. *Qualitative Research* 2019;20(4):485-515. doi: 10.1177/1468794119881985

64. Sanson A, Prior, M., Garino, E., Oberklaid, F., Sewell, J. The Structure of Infant Temperament: Factor Analysis of the Revised Infant Temperament Questionnaire. *Infant Behavior and Development* 1987;10:97-104.

65. Squires J, Bricker D, Twombly E. The ASQ: SE user's guide: For the Ages & Stages Questionnaires: Social-emotional: Paul H Brookes Publishing 2002.

66. Goodman R. The Strengths and Difficulties Questionnaire: a research note. *Journal of child psychology and psychiatry* 1997;38(5):581-86.

67. Waters E, Salmon L, Wake M, et al. The Child Health Questionnaire in Australia: reliability, validity and population means. *Australian and New Zealand journal of public health* 2000;24(2):207-10.

68. Kessler RC, Barker, P.R., Colpe, L.J., Epstein, J.F., Gfroerer, J.C., Hiripi, E., Howes, M.J, Normand, S-L.T., Manderscheid, R.W., Walters, E.E., Zaslavsky, A.M.,. Screening for serious mental illness in the general population. *Archives of General Psychiatry* 2003;60(2):184-89.

69. Ware JE, Kosinski M, Keller SD. A 12-Item Short-Form Health Survey: Construction of Scales and Preliminary Tests of Reliability and Validity. *Medical Care* 1996;34(3):220-33.

70. Zubrick S, Lucas N, Westrupp E, et al. Parenting measures in the Longitudinal Study of Australian Children: construct validity and measurement quality, Waves 1 to 4. Canberra: Department of Social Services 2014.

71. EuroQol. EQ-HWB: EuroQol; 2021 [Available from: https://euroqol.org/blog/eq-hwb/ accessed 27/05 2021.

72. Australian Bureau of Statistics (ABS). Patient Experience Survey Canberra: ABS; 2010 [Available from: https://www.abs.gov.au/statistics/health/health-services/patient-experiences-australia-summary-findings accessed 02/06 2021.

For peer review only - http://bmjopen.bmj.com/site/about/guidelines.xhtml

73. International Wellbeing Group. Personal Wellbeing Index: . 5th Edition ed. Melbourne: Australian Centre on Quality of Life, Deakin University, 2013.

74. Lewis JM. A network approach for researching partnerships in health. *Aust New Zealand Health Policy* 2005;2:22. doi: 10.1186/1743-8462-2-22

75. Provan K, Nakama, L., Veazie, MA.,Teufel-Shone, NI., & Huddleston, C.,. Building Community Capacity Around Chronic Disease Services Through a Collaborative Interorganizational Network. *Health Education & Behavior* 2003;30(6):646-62.

76. Provan K, Harvey, J., Guernsey de Zapien, J., . Network structure and attitudes toward collaboration in a community partnership for diabetes control on the US-Mexico border. *Journal of Health Organization and Management* 2005;19(6):504.

77. Valente TW, Pitts SR. An Appraisal of Social Network Theory and Analysis as Applied to Public Health: Challenges and Opportunities. *Annu Rev Public Health* 2016 doi: 10.1146/annurev-publhealth-031816-044528

78. StataCorp. Stata Statistical Software: Release 17. Texas, US: StataCorp LLC, 2021.

79. R Core Team. R: A language and environment for statistical computing. Vienna, Austria: R Foundation for Statistical Computing, 2020.

80. Borgatti SP, Everett, M.G., Freeman, L.C.,. Ucinet for Windows: Software for Social Network Analysis. Harvard, MA: Analytic Technologies, 2002.

81. NVivo qualitative data analysis software [program]. Release 1.0 version, 2020.

82. Braun V, Clarke V. Reflecting on reflexive thematic analysis. *Qualitative Research in Sport, Exercise and Health* 2019;11(4):589-97. doi: 10.1080/2159676X.2019.1628806

83. Patton M. Enhancing the Quality and Credibility of Qualitative Analysis. *Health Services Research* 1999;34(5):1189-208.

84. Fielding NG. Triangulation and Mixed Methods Designs: Data Integration With New Research Technologies. *Journal of Mixed Methods Research* 2012;6(2):124-36. doi: 10.1177/1558689812437101

85. Boaz A, Hanney S, Borst R, et al. How to engage stakeholders in research: design principles to support improvement. *Health Res Policy Syst* 2018;16(1):60. doi: 10.1186/s12961-018-0337-6 [published Online First: 2018/07/13]

Authors contributions

HH, SG and JE are Chief Investigators responsible for conceptual development, study design and manuscript preparation. TH was involved in concept development, study design

For peer review only - http://bmjopen.bmj.com/site/about/guidelines.xhtml

and drafted the manuscript. All authors were involved in concept development, study design and manuscript preparation, and approved the final manuscript.

Funding statement

This research is supported by the Australian National Health and Medical Research Council and Beyond Blue grant number 1153419. Murdoch Children's Research Institute is supported by the Victorian Government's Operational Infrastructure Support Program. Professor Harriet Hiscock is supported by an NHMRC Practitioner Fellowship 1136222. Professor Sharon Goldfeld is supported by an NHMRC Practitioner Fellowship 1155290. Professor Jane Fisher holds the Finkel Chair in Global Health which is supported by the Finkel Family Foundation.

Competing interests statement

There are no competing interests to declare.

Patient consent for publication

Not required.

For peer review only

For peer review only - http://bmjopen.bmj.com/site/about/guidelines.xhtml

Integrated Child and Family Hub models for detecting and responding to family adversity: protocol for a mixed-methods evaluation in two sites.

Journal:	*BMJ Open*
Manuscript ID	bmjopen-2021-055431.R1
Article Type:	Protocol
Date Submitted by the Author:	12-Apr-2022
Complete List of Authors:	Hall, Tess; Murdoch Childrens Research Institute,<br>Goldfeld, Sharon; Murdoch Children's Research Institute<br>Loftus, Hayley; Murdoch Children’s Research Institute, Health Services; Royal Children’s Hospital, Health Services<br>Honisett, Suzy; Murdoch Children's Research Institute<br>Liu, Hueiming; The George Institute for Global Health,<br>De Souza, Denise ; Torrens University Australia - Fitzroy Campus<br>Bailey, Cate; The University of Melbourne School of Population and Global Health<br>Reupert, Andrea; Monash University, Education<br>Yap, Marie; Monash University,<br>Eapen, Valsamma; South Western Sydney Local Health District, ICAMHS; University of New South Wales, School of Psychiatry<br>Haslam, Ric; The Royal Children's Hospital Melbourne<br>Sanci, Lena; University of Melbourne, Department of General Practice<br>Fisher, Jane; Monash University, Jean Hailes Research Unit, School of Public Health and Preventative Medicine<br>Eastwood, John; The University of Sydney,<br>Mukumbang, Ferdinand; University of the Western Cape, School of Public Health; Ferdinand C. Mukumbang,<br>Loveday, Sarah; Murdoch Childrens Research Institute<br>Jones, Renee; Murdoch Childrens Research Institute<br>Constable, Leanne; Murdoch Childrens Research Institute<br>Forell, Suzie; Health Justice Australia<br>Morris, Zoe; Monash University<br>Montgomery, Alicia; Marrickville Health Centre<br>Pringle, Glenn; IPC Health<br>Dalziel, Kim; The University of Melbourne, School of Population and Global Health<br>Hiscock, Harriet; The Royal Children's Hospital Melbourne, Centre for Community Child Health
**Primary Subject Heading**:	Health services research
Secondary Subject Heading:	Paediatrics, Mental health
Keywords:	Protocols & guidelines < HEALTH SERVICES ADMINISTRATION & MANAGEMENT, MENTAL HEALTH, Community child health < PAEDIATRICS, PRIMARY CARE, Child & adolescent psychiatry <

	PSYCHIATRY, PUBLIC HEALTH

SCHOLARONE™
Manuscripts

Integrated Child and Family Hub models for detecting and responding to family adversity: protocol for a mixed-methods evaluation in two sites.

For peer review only

Correspondence to Professor Harriet Hiscock

Murdoch Children's Research Institute: 50 Flemington Rd, Parkville, Victoria, Australia 3052

harriet.hiscock@rch.org.au

Author name	Department	Organisation	City, State, Country
Teresa Hall	Centre for Community Child Health	Murdoch Children's Research Institute	Parkville, Victoria, Australia
Sharon Goldfeld	Centre for Community Child Health	Murdoch Children's Research Institute	Parkville, Victoria, Australia
Hayley Loftus	Centre for Community Child Health	Murdoch Children's Research Institute	Parkville, Victoria, Australia
Suzy Honisett	Centre for Community Child Health	Murdoch Children's Research Institute	Parkville, Victoria, Australia
Huei Ming Liu	Sydney Institute for Women, Children and their families[a]<br>The George Institute for Global Health[b]	Sydney Local Health District[a]<br>University of New South Wales[b]	Marrickville, New South Wales, Australia
Denise De Souza	Centre for Research in Education and Sustainability	Torrens University	Melbourne City, Victoria, Australia
Cate Bailey	Melbourne School of Population and Global Health	University of Melbourne	Parkville, Victoria, Australia
Andrea Reupert	Krongold Clinic	Monash University	Notting Hill, Victoria, Australia
Marie Yap	School of Psychological Sciences	Monash University	Clayton, Victoria, Australia

Valsamma Eapen	Infant Child and Adolescent Psychiatry	University of New South Wales	Sydney, New South Wales, Australia
Ric Haslam	Department of Paediatrics	University of Melbourne	Parkville, Victoria, Australia
Lena Sanci	Department of General Practice	University of Melbourne	Parkville, Victoria, Australia
Jane Fisher	Global Health and Women's Health	Monash University	Melbourne, Victoria, Australia
John Eastwood	Clinical Services Integration and Population Health	Sydney Local Health District	Camperdown, New South Wales, Australia
Ferdinand C Mukumbang	Department of Social Work	University of the Western Cape	Cape Town, South Africa
Sarah Loveday	Centre for Community Child Health	Murdoch Children's Research Institute	Parkville, Victoria, Australia
Renee Jones	Centre for Community Child Health	Murdoch Children's Research Institute	Parkville, Victoria, Australia
Leanne Constable	Centre for Community Child Health	Murdoch Children's Research Institute	Parkville, Victoria, Australia
Suzie Forell	[no department for [a]]<br><br>School of Law[b]	Health Justice Australia[a]<br>University of New South Wales[b]	Darlinghurst, Sydney, Australia
Zoe Morris	Department of Education	Monash University	Clayton, Victoria, Australia
Alicia Montgomery	Marrickville Health Centre	Sydney Local Health District	Marrickville, New South Wales, Australia
Glenn Pringle	Innovation and Community Care	IPC Health	Wyndham Vale, Victoria, Australia
Kim Dalziel	Melbourne School of Population and Global Health	University of Melbourne	Parkville, Victoria, Australia
Harriet Hiscock	Centre for Community Child Health	Murdoch Children's Research Institute	Parkville, Victoria, Australia

Abstract

Introduction: Integrated community health care Hubs may offer a 'one stop shop' for service users with complex health and social needs, and more efficiently use service resources. Various policy imperatives exist to implement Hub models of care, however, there is a dearth of research specifically evaluating Hubs targeted at families experiencing adversity. To contribute to building this evidence, we propose to co-design, test and evaluate integrated Hub models of care in two Australian community health services in low socioeconomic areas that serve families experiencing adversity: Wyndham Vale in Victoria and Marrickville in NSW.

For peer review only

For peer review only - http://bmjopen.bmj.com/site/about/guidelines.xhtml

Methods and analysis: This multi-site convergent mixed-methods study will run over three phases to (1) develop the initial Hub program theory through formative research; (2) test and then (3) refine the Hub theory using empirical data. Phase 1 involves co-design of each Hub with caregivers, community members and practitioners. Phase 2 utilises caregiver and Hub practitioner surveys at baseline, and 6- and 12-months after Hub implementation, and in-depth interviews at 12-months. Two stakeholder groups will be recruited: caregivers (n = 100-200 per site) and Hub practitioners (n = 20-30 per site). The intervention is a co-located Hub providing health, social, legal and community services with no comparator. The primary outcomes are caregiver-reported: (i) identification of, (ii) interventions received and/or (iii) referrals received for adversity from Hub practitioners. The study also assesses child, caregiver, practitioner, and system outcomes including mental health, parenting, quality of life, care experience and service linkages. Primary and secondary outcomes will be assessed by examining change in proportions/means from baseline to 6-months, from 6- to 12-months, and from baseline to 12-months. Service linkages will be analysed using social network analysis. Costs of Hub implementation and a health economics analysis of unmet need will be conducted. Thematic analysis will be employed to analyse qualitative data.

Ethics and dissemination: Royal Children's Hospital and Sydney Local Health District ethics committees have approved the study (HREC/ 62866/RCHM-2020). Participants and stakeholders will receive results through meetings, presentations and publications.

Trial registration number: ISRCTN55495932

Strengths and weaknesses of this study

- The 'Child and Family Hub' study is the first to our knowledge to co-design, test and evaluate two integrated Hub models of care for improving the detection of and response to family adversity for children aged 0-8 years.
- Implementation and evaluation in two community health services across two Australian states will maximise the generalisability of findings for different community and service contexts.
- The interdisciplinary conceptual framework underpinning this study strengthens its empirical robustness through synthesis of approaches from many disciplines to capture the complexity of the Hub models.

For peer review only - http://bmjopen.bmj.com/site/about/guidelines.xhtml

- The study has no comparison group which means that any observed changes for primary and secondary outcomes cannot be directly attributed to the Hub models of care.
- The English-language inclusion criterion for participants limits our ability to engage culturally and linguistically diverse caregiver and practitioners.

Introduction

Family adversity and its negative impact on mental health

Interventions aimed at reducing or preventing the impact of adversity on children could avert a substantial proportion of the population burden of mental illness, and build on evidence demonstrating the health and economic benefits of investing early in life.[1] Family adversity includes a range of adverse childhood experiences (ACEs) such as childhood maltreatment (e.g., physical, verbal or sexual abuse), household dysfunction (e.g., parental mental illness, family substance abuse), community dysfunction (e.g., witnessing physical violence, discrimination), peer dysfunction (e.g., stealing, bullying) and socio-economic deprivation.[2] While family adversity can negatively impact a range of physical and mental health outcomes, its negative effects on children's mental health are well established, increasing the risk of anxiety, depression and suicidality in childhood and across the life course. [3-8] Further, exposure to multiple ACEs predicts greater odds of poorer mental versus physical health outcomes in adult life.[9] Experiences of family adversity also negatively affect child neurological and physiological development and educational outcomes,[10] which in turn lead to poor life-long health and mental health outcomes.[11]

Detection and response to family adversity

As part of an Australian Centre of Research Excellence (CRE) in Childhood Adversity and Mental Health, we will develop, test and evaluate an approach to improving the detection of and response to adversity in children aged 0-8 years and their families. Effective detection of adversity is crucial to the provision of appropriate, timely and evidence-based responses to the effects of adversity. However, major service barriers exist to the detection of, and responses to, adversity, including service time pressures, a lack of practitioner time, training, and confidence in how to effectively discuss adversities, and practitioners' concerns about causing harm.[5 12-14] Family-level barriers also inhibit service access, including cognitive (i.e., knowledge, awareness of services, etc), psychological (i.e., fear, shame and distrust of

For peer review only - http://bmjopen.bmj.com/site/about/guidelines.xhtml

services), financial, and structural barriers (i.e., service availability, lengthy wait times, transport, etc).[15-17] These service access barriers are amplified for families experiencing multiple adversities who require supports from health, social, education and community service systems. These systems are often siloed which is likely to contribute to the low utilisation of services by these families.[18 19] Hence, effective detection of, and responses to, adversity require intervention across multiple strata of intersectoral service systems.

Integrated care for families experiencing adversity

Integrated care has the potential to overcome service fragmentation and respond to a range of holistic health and social needs for families and their children experiencing adversity. Integration is defined as care that: "*connect[s] the healthcare system (acute, primary medical and skilled) with other human service systems (e.g., long-term care, education and vocational and housing services) to improve outcomes (clinical, satisfaction and efficiency)".*[20] Integrated care initiatives can increase uptake and ongoing engagement with child health services [21 22], improve child mental health outcomes[23 24] and offer a cost-effective and acceptable service response for families.[25 26] Integrated community health care Hubs have recently gained traction globally, driven by their appeal to offer a 'one stop shop' for service users with complex health and social needs, and to efficiently use service resources.[27] While there is no single definition of a Hub, the term is often used to describe a centralised service that offers a range of co-located, integrated services from multiple sectors, with linkages to external services for community-based supports.[27 28] Emerging evidence exists for outcomes of Hub models of care. For example, the Healthy Homes and Neighbourhoods (HHAN) initiative in Sydney, New South Wales (NSW), is a multi-component cross-agency care coordination network with a centralised Hub nested within a broader place-based initiative and has been shown to increase service access for families experiencing adversity.[29 30]

Various policy imperatives have been established to implement and evaluate Hub models of care for child and family mental health in Australia. These include the 2021 Royal Commission into Victoria's Mental Health System (recommending implementation and evaluation of "*infant, child and family health and wellbeing multidisciplinary community-based hubs*"[31]) and the 2021 National Children's Mental Health and Wellbeing Strategy.[32] The increased rates of child mental health problems resulting from the COVID-19 pandemic underscore the need for accessible, effective mental health support in communities.[33]

For peer review only - http://bmjopen.bmj.com/site/about/guidelines.xhtml

However, there is a dearth of research specifically evaluating Hub models of integrated care for families experiencing adversity with a particular focus on child mental health. Extant research also does not specify when and how integration of care occurs within Hubs across the layers of a service ecological system (i.e., clinical, professional, organisational and systems integration[31]), and which types of integration in co-located Hub models drive observed changes for children and families.

The current study

To contribute to building this evidence, we propose to co-design, test and evaluate integrated Hub models of care in two Australian community health care services in low socioeconomic, metropolitan areas that serve families experiencing adversity, i.e., Wyndham Vale in Victoria and Marrickville in NSW. Twenty-three percent of children in Wyndham and 14.6% of children in Marrickville are developmentally vulnerable in one or more domains of the Australian Early Development Census [34]. In this study, we define a Hub as a co-located service providing an intersectoral health, social, legal and community response to family adversity. The Wyndham Vale site is a Hub in a local government area and the Marrickville site is a Hub nested within a broader place-based initiative. Co-design of a Hub at each site will involve caregivers, community members and service providers. The evidence generated from this study is crucial for increasing the conceptual precision around integrated Hub models of care and in addressing the evidence gap on their effectiveness for detecting and responding to family adversity and promoting positive child and caregiver mental health.

Study aims and objectives

Aim

1. To co-design, test and evaluate integrated Child and Family Hub models for detecting and responding to family adversity in children aged 0-8 years and their families in Wyndham Vale, Victoria, and Marrickville, NSW, and
2. To formulate a realist-informed program theory of how, why, for whom and under what conditions the Hubs work to detect and respond to family adversity.

Objectives

1. To assess the impact of the Hub models on:

   Primary outcomes

   1.1. caregiver-reported (i) identification of, (ii) interventions received and/or (iii) referrals received for adversity from Hub practitioners,

For peer review only - http://bmjopen.bmj.com/site/about/guidelines.xhtml

Secondary outcomes

1.2. Hub practitioner-reported (i) identification of, (ii) interventions delivered and/or (iii) referrals provided for adversity,
1.3. caregiver-reported uptake of referrals,
1.4. child and caregiver mental health,
1.5. infant temperament,
1.6. child and caregiver global health,
1.7. caregiver parenting,
1.8. caregiver quality of life,
1.9. caregiver personal wellbeing outcomes,
1.10. caregiver experience of care,
1.11. practitioner experience including confidence and competence in detecting and responding to adversity,
1.12. linkages between intersectoral Hub practitioners, and

2. To determine the costs of implementing the Hub models, and
3. To determine the extent that the Hub models are providing the intended service option (i.e., acceptability, feasibility, and fidelity to the initial Hub program theory).

Reporting in this protocol is based on the Strengthening the Reporting of Observational Studies in Epidemiology (STROBE) Statement. [35]

Methods and analysis

This study is a multi-site implementation and evaluation project that employs a convergent mixed-methods design conducted over three research phases displayed in Figure 1 below. Quantitative and qualitative data will be collected in parallel and integrated during data analysis and interpretation.[36]

Interdisciplinary conceptual framework

The study is underpinned by an interdisciplinary conceptual framework drawn from implementation science, critical realism, improvement science and collaborative co-design paradigms.[37-44] Interdisciplinary research strengthens empirical robustness through synthesis of approaches from many disciplines, and has been shown to increase the uptake of research into policy and practice.[45] The approach and rationale for the interdisciplinary framework in order of importance for this study are:

For peer review only - http://bmjopen.bmj.com/site/about/guidelines.xhtml

- *Implementation science*: Given the focus of our inquiry on complex Hub interventions, key implementation science frameworks form the basis of our process and outcomes evaluation, including our framing and synthesis of context using the Consolidated Framework for Implementation Research (CFIR)[42].
- *Critical realism and realist evaluation*: We draw upon elements of critical realism and realist evaluation, a form of theory-driven evaluation, to theorise and examine 'what works, for whom, under what circumstances, and how'.[46-49] Realist evaluation assumes that interactions between context and intervention mechanisms produce the outcomes of an intervention.[47 48 50 51] We focus our inquiry on the key context-intervention-mechanism-outcome (CIMO) configurations discussed below. We complement realist evaluation methods with critical realism[52 53] to understand the historical aspects of each locality and how this history influences the design and workings of each new Hub. This overcomes a key shortcoming of realist evaluation, that is, under theorising the multiple levels of social reality surrounding each Hub.[54 55]
- *Improvement science*: We use improvement science methods to implement agile learning and testing cycles that will iterate the Hub models and embed learning systems into practice.[43]
- *Collaborative co-design:* We use these principles and methods to co-design the Hub models and promote stakeholder engagement throughout the research process.

Figure 1 displays the three study phases based on Pawson and Tilley's [46] realist evaluation framework: (1) Development of the initial Hub program theory through formative research; (2) Testing of the initial Hub theory using empirical data; and (3) Refinement of the initial Hub theory.

[insert Figure 1]

Study phase 1: Development of initial Hub program theory through formative research

The initial Hub program theory was developed through an intensive formative research phase during which we collected contextual information and co-designed the Hub models with intersectoral (e.g., health, social, legal and education) practitioners and caregivers from each local community. This phase occurred from October 2020 to June 2021 in Victoria and January to August 2021 in NSW. First, a historical and current needs assessment of the

For peer review only - http://bmjopen.bmj.com/site/about/guidelines.xhtml

health, social, legal and community contexts of each site was conducted using qualitative data from online group discussions, individual interviews and meetings with key stakeholders including intersectoral practitioners (i.e., from health, social, legal and community sectors) and caregivers. We used CFIR[42] to capture and synthesise these data into barriers and enablers across the outer setting, inner setting, individual level, process and intervention components.

Second, we reviewed the HHAN program theories and other relevant realist literature focused on integrated care for children and families experiencing adversity[23 30 56-59] to identify key CIMO configurations relevant to the Hubs. HHAN provided a useful starting point because our study builds on the HHAN model of care. For pragmatic reasons, we focus the process and outcome evaluation in this study on three key mechanisms and associated CIMO configurations in the initial program theory. Namely, (1) trust between caregivers and the service/practitioners, and between practitioners, (2) knowledge acquisition and motivation of practitioners, and (3) perceived benefits of collaboration by practitioners. Figure 2 displays the initial realist-informed program theory. We then formulated the logic model displayed in Figure 3 proposing how the Hub models will be investigated and are theorised to contribute to outcomes. The logic model includes how immediate outcomes, i.e., increased detection and response to adversity are hypothesized to lead to our intended outcomes, including increased uptake of services and improved child and caregiver mental health and caregiver quality of life.

Third, the Hub models were then developed and refined through a collaborative co-design process with intersectoral practitioners, caregivers and children at each site. The co-design process focused on the client journey through each proposed Hub and the workforce capacities and infrastructure needed to support implementation of the Hub. In Wyndham Vale, the Hub model was co-designed through an intensive 10-week series of co-design workshops and consultations with local caregivers and practitioners using human-centred design processes.[37-39] In Marrickville, the Hub model will be co-designed through focus group discussions and workshops employing the Nominal Group Technique consensus method [60] to prioritise and develop implementation strategies. Different approaches to co-design are adopted that reflect the capacity, capabilities and preferences for engagement of the research team and co-design partners at each site.

The theorised Hub models comprise:

For peer review only - http://bmjopen.bmj.com/site/about/guidelines.xhtml

- *Family and community partnerships*: intentional creation and strengthening of connections between the Hub and caregivers, community groups and individuals. These partnerships also involve Hub practitioners working in partnership with families during clinical practice.
- *Entry to the Hub:* a 'no wrong door' approach in which caregivers are safely engaged in a conversation about adversity and provided with any necessary support and/or referrals regardless of how they enter the Hub.
- *Workforce development*: workforce capacity building and training of Hub practitioners to engage families in a safe and respectful conversation to identify adversities and connect families to relevant support.
- *Case-based discussions:* monthly professional development with intersectoral Hub practitioners to embed training learnings into practice and facilitate between-practitioner referrals (i.e., 'warm referrals').
- *Referral pathways into and out of the Hub*: systematic mapping of available health, community and social sector supports and services in the local area, linked to training of Hub practitioners to use this information in their practice.
- *Care navigation*: In Wyndham Vale, a Wellbeing Coordinator will support caregivers to identify the holistic needs of their child and/or family and assist them to navigate relevant services and supports in the community, social and health sectors. In Marrickville, care navigation is likely to include a Wellbeing Coordinator or a virtual application for caregivers that provides a risk stratification and recommendations of relevant services.

[insert Figure 2]

[insert Figure 3]

Study phase 2: Testing of initial Hub theory using empirical data

We will empirically test the initial program theory for the Hubs through a mixed-methods process with 6- and 12-months outcomes evaluation. The evaluation aims to assess for whom, how and why the Hub models had an impact (if any) in the two different contexts of Wyndham Vale and Marrickville across the child, caregiver, practitioner, and system level outcomes specified in Figures 2 and 3, Table 1 and 'Outcomes Assessment' below.

For peer review only - http://bmjopen.bmj.com/site/about/guidelines.xhtml

Outcome evaluation

Design

The study is a mixed-methods repeated measures evaluation that utilises caregiver and Hub practitioner surveys at baseline, 6- and 12-months after Hub implementation begins and in-depth interviews at 12-months.

Participants

We aim to recruit two stakeholder groups: caregivers (*n* = 100-200 per site) and Hub practitioners (*n* = 20-30 per site).

Caregivers

Potential caregiver participants are those who: (i) care for a child aged 0-8 years, including women who are pregnant, (ii) access any of the universal or specialist services provided in the Hub, such as General Practitioners (GPs), paediatricians, lawyers, etc, and (iii) can understand written or spoken English-language. The English-language criterion is a limitation of our study; however, funding is not available to provide interpreters. Furthermore, culturally and linguistically diverse persons participated in an informed and meaningful way in phone-based or in-person surveys for HHAN (personal communication Prof Eastwood).

Given the preventative focus of this study, caregivers will not be screened for adverse experiences as an inclusion criterion. Instead, caregivers will report the frequency of fifteen adversity types in the baseline survey. These adversity types are derived from the Parent Engagement Resource[61] and include challenges with: social support, finances, housing, employment, family physical health, parent mental health, parenting, child neglect, alcohol and substance use, family relationships, family violence, child abuse, visa and immigration issues, crime issues and discrimination.

Hub practitioners

Potential practitioner participants are those who: (i) work in any of the intersectoral services provided as part of each Hub, including GPs, maternal and child health nurses, paediatricians, allied health professionals, social workers, lawyers, and (ii) can understand written or spoken English-language.

For peer review only - http://bmjopen.bmj.com/site/about/guidelines.xhtml

Sample size calculation

As this study is testing the Hub models using a non-experimental design, we have not conducted a formal sample size calculation. However, the sample size of 200-400 caregivers is likely to provide rich data on the primary and secondary outcomes.

Recruitment and consent

The study is designed to accommodate a range of participants, many of whom may have complex life circumstances.[62] Figure 4 presents the caregiver participant study flow. We will recruit caregivers from the waiting rooms of each Hub and through Hub practitioners who will ask their clients' permission for the research team to contact them. Researchers will approach potential caregiver participants (in the waiting room or on the telephone), show and/or email them an informational video and study information pack (including a written Caregiver Participant Information Consent Form, PICF), and invite them to take part in the study. Participants can provide informed consent verbally on the phone or in-person, or in writing by clicking through the online consent form preceding the survey. Caregivers will be provided with a $25 honorarium for each survey and/or interview they complete.

We will invite practitioners working in each Hub who have been identified and recruited by the study team or health centre managers. We will send an invitation email to potential practitioner participants with the Practitioner PICF attached before they attend the workforce training. Practitioner participants will provide written (online or hard copy) informed consent prior to completing the baseline survey.

[insert Figure 4]

Outcome assessment

The study will assess the child, caregiver, practitioner, and systems outcomes summarised in Table 1.

Data collection

Caregiver and Hub practitioner surveys

The Caregiver and Hub practitioner baseline and outcome surveys consisting of the measures outlined in Table 1 and available in Supplementary File 1 will be built in REDCap[63] hosted by Murdoch Children's Research Institute. After completing an informed consent process, caregivers can complete the baseline survey: (i) in paper form, (ii) over the telephone with a

For peer review only - http://bmjopen.bmj.com/site/about/guidelines.xhtml

researcher or (iii) online. Practitioners will complete the practitioner baseline survey in paper form or online via REDCap. The practitioner survey will be sent towards the end of baseline data collection to minimise the potential impact of the survey on clinical practice prior to the Hub testing period. Caregiver and practitioner participants will be contacted by telephone, email or text message by the research team 6- and 12-months after the Hub implementation begins and invited to complete the follow up survey.

Caregiver and Hub practitioner interviews

Twelve-months after Hub implementation begins, we will conduct realist-informed semi-structured interviews with 18-24 caregivers and 5-10 Hub practitioners per site.[64] We will purposively sample participants to capture caregivers from diverse cultural backgrounds, experiences of adversities and varying levels of engagement with intersectoral services. These realist-informed interviews will enhance the testing of the initial program theory to triangulate with the findings from the quantitative measures. The interviews aim to uncover (i) the mechanisms and contextual elements relevant to the Hub models (to inform theory development), (ii) acceptability and feasibility of the Hub, (iii) their experience of being asked about adversity, the care offered to them, and the process of referrals including barriers and enablers to uptake (caregivers only), and (iv) confidence and competence in detecting and supporting families experiencing adversity (practitioners only).

For peer review only

For peer review only - http://bmjopen.bmj.com/site/about/guidelines.xhtml

Table 1 Primary and secondary outcomes

PRIMARY OUTCOMES		BASELINE ASSESSMENT		6- AND 12-MONTHS POST HUB IMPLEMENTATION BEGINS	
		Caregiver survey	Practitioner survey	Caregiver survey	Practitioner survey
Caregiver					
**Identification of adversity**	Increase in the proportion of caregivers who report being asked by a service provider about adversity in the past six months	X		X	
**Intervention for adversity**	Increase in the proportion of caregivers who report spending extra time with or receiving an intervention from a Hub service provider for adversity in the past six months	X		X	
**Referrals for adversity**	Increase in the proportion of caregivers who report receiving a referral to an intersectoral service for adversity in the past six months	X		X	
SECONDARY OUTCOMES					
Child					
**Infant temperament**	Increase in the proportion of caregivers who report their infant is easier/much easier than average; assessed through single caregiver-reported item on infant temperament; has a moderate correlation (r=0.51) with the Easy-Difficult Scale (EDS) of Australian version short form of Revised Infant Temperament Questionnaire[65]; completed for one child* aged 0-8 in each family 0 to < 2 years	X		X	
**Child mental health**	Decrease in the mean scores for caregiver-reported internalising or externalising symptoms for their child. Completed for one child* in each family. For children aged 0 to < 2 years:	X		X	

For peer review only - http://bmjopen.bmj.com/site/about/guidelines.xhtml

	Ages & Stages Questionnaire Social-Emotional Second Edition (ASQ-SE2)[66^]<br>For children aged ≥ 2 to 8 years: Strengths & Difficulties Questionnaire [67]				
**Global health**	Increase in the mean scores of caregiver-reported general child health; assessed through single item (GHQ-S1) from Child Health Questionnaire [68]; completed for one child* aged 0-8 in each family	X		X	
Caregiver					
**Uptake of referrals**	Increase in the proportion of caregivers who report uptake of referrals to other services in the past six months.	X		X	
**Mental health**	Decrease in the mean scores of caregiver-reported psychological distress as assessed by Kessler Psychological Distress Scale 6 (K6); 6-item [69]	X		X	
**Global health**	Increase in means scores of caregiver-reported general health; assessed through single item of the Short Form Health Survey (SF-12)[70]	X		X	
**Parental warmth, parenting hostility and efficacy**	Increase in mean scores of parental warmth and efficacy; decrease in mean scores on parenting hostility. Three self-report subscales drawn from the Longitudinal Study of Australian Children (LSAC), Australia's first nationally representative longitudinal study of child development which will allow for national comparisons of the study data [71]; assessing parental warmth, parenting hostility and efficacy. Parental warmth (6-items), parenting hostility (5-items) and parenting efficacy (4-items)	X		X	
**Quality of life**	Increase in mean caregiver quality of life scores calculated from the	X		X	

For peer review only

For peer review only - http://bmjopen.bmj.com/site/about/guidelines.xhtml

	EuroQol Health and Wellbeing Instrument Short Form (EQ-HWB-S) [72]				
**Caregiver experience**	Caregiver reported acceptability and feasibility of the Hub; Increase in the proportion of caregivers who report their satisfaction with Hub care as measured by 3 items from the Australia Bureau of Statistics Patient Experiences in Australia Survey [73]	X		X	
**Personal wellbeing**	Increase in mean scores of caregiver-reported personal wellbeing outcomes measured by the Personal Wellbeing Index; 7 items [74]	X		X	
Practitioner					
**Identification of adversity**	Increase in the proportion of practitioners who report asking about adversity in the past six months		X		X
**Intervention for adversity**	Increase in the proportion of practitioners who report spending extra time or providing an intervention for adversity in the past six months		X		X
**Referrals for adversity**	Increase in the proportion of practitioners who report referring caregivers to an intersectoral service for adversity in the past six months		X		X
**Practitioner experience**	Practitioner reported acceptability and feasibility of the Hub; Increase in the proportion of practitioners who report feeling confident and competent to detect and support families experiencing adversity		X		X
System level					
**Strength and structure of intersectoral service linkages**	Increase in the number and strength of service linkages between Hub practitioners as assessed through social network analysis (SNA) indicators for network density, degree, centrality and betweenness, and map of linkages between Hub practitioners based on i) contact, (ii) referrals to, (iii) referrals from, and (iv) quality of the		X		X

For peer review only - http://bmjopen.bmj.com/site/about/guidelines.xhtml

	relationship[75-78]				
**Health economics outcomes**	Costs of implementation of the Hub models, caregiver-reported intersectoral service usage and value of unmet need	X		X	

* Caregivers with more than one child will respond to questions pertaining to one child in their family based on the child they are most concerned about.

^ The ASQ-SE is limited by its design as a screening tool that may not be a sensitive outcome measure. The measure is used in this study because it more directly measures mental health and wellbeing than the ASQ.

Quantitative data analysis

Statistical analyses will be conducted using STATA or R statistical software packages.[79 80] The baseline characteristics of the caregivers, children and practitioners will be described using the mean, median and interquartile range for continuous data and proportions for categorical data. Primary and secondary outcomes will be assessed by examining change in proportions/means from baseline to 6-, from 6- to 12- and from baseline to 12-months. We will also conduct linear regression analyses to assess the association between each caregiver-reported primary outcome at 6- and 12-months with the outcomes of child and caregiver mental health and caregiver quality of life, at 6 and 12-months, respectively.

Quantitative measures of the strength and structure of service linkages between Hub practitioners will be analysed using social network analysis (SNA) software package UCINET.[81] SNA is a complex systems discipline and quantitative methodology widely used to measure networked relationships between organisations and individual actors in health service and policy settings.[75-78] UCINET[81] will calculate indicators (i.e., density, degree, centrality and betweenness) for the network and each Hub practitioner based on: i) contact, (ii) referrals to and from, and (iii) quality of the relationship. These indicators and analytical approach are recommended for descriptive social network analysis.[75-78]

Health economics analysis

Costs of the Hubs will be identified, including practitioner training, administration for referrals, personnel to oversee clinical implementation and other relevant costs. Downstream costs of services and potential cost-offsets will be collected and analysed to obtain the cost per additional child/or caregiver attending the Hub. Quality of life will be measured using the carer-specific measure (EQ-HWB-S[72]). An analysis of the value of unmet need will be conducted by determining whether (i) the child and family's needs were met, (ii) no needs, or

For peer review only

For peer review only - http://bmjopen.bmj.com/site/about/guidelines.xhtml

(iii) unmet needs using the ASQ or SDQ to determine perceived need compared to the level of service use.

Qualitative data analysis

Individual interviews will be transcribed verbatim by a transcription service and imported into N-Vivo Release 1.0 [82] to assist in the process of analysis. Experienced qualitative researchers will employ Braun and Clarke's six-stage reflexive thematic analysis [83] to analyse the qualitative interview data. In line with the critical realist philosophy underpinning this study, we will employ inductive coding and deductive (*a priori*) coding based on the CIMO configurations for the Hub.

Process evaluation and quality improvement cycles

We will conduct a process evaluation using multiple data sources, including routinely collected data (e.g., attendance rates at training and visits to the Wellbeing Coordinator) and observation field notes of Hub practitioner clinical practice, the training sessions and the case-based discussions. The process evaluation examines the acceptability, feasibility, and fidelity of implementation for each Hub component shown in the initial Hub program theory in Figure 2. The process evaluation will also include realist analytical methods in which observed outcomes are explained by looking into the mechanisms and contextual elements contributing to the focus CIMOs for this study.

We will also conduct improvement cycles guided by the Model for Improvement.[43] Small improvement teams will be formed at each site who will establish their specific improvement aims and measurements. Once the aims are agreed, the team will undertake short learning cycles via Plan-Do-Study-Act (PDSA) cycles.[43] Each PDSA cycle begins by articulating: the change and recording predictions about what we expect to happen (Plan); attempting to make the change and documenting what happened (Do); comparing the results to the predictions (Study); and then deciding on what to do next (Act). By the end of the project, learning cycles will be embedded as part of routine Hub service delivery, making the Hub more likely to be sustained and used.[41] To maximise rigor, we will triangulate data collected through the process evaluation and PDSA cycles with outcome measures collected through the 6- and 12-month surveys.[84]

For peer review only - http://bmjopen.bmj.com/site/about/guidelines.xhtml

Study phase 3: Refinement of initial Hub program theory

We will use the process and outcome evaluation data to refine the initial Hub program theory. Further testing will be undertaken by explicitly seeking to confirm or contradict theories throughout the PDSA cycles, in-depth interviews and knowledge translation activities (e.g., forums, workshops, and webinars). A key strength of our study is the assessment of outcomes across multiple levels of the Hub intervention (i.e., child, family, practitioner, service and system levels). The triangulation of multiple data sources to evaluate the implementation of the Hub model serves to maximise the study's rigour and confidence in our findings.[84 85]

Patient and public involvement

Caregivers and practitioners were actively involved in the co-design of the Hub models and are members of local advisory groups at each site which oversee the study design, recruitment, piloting of instruments, interpretation and dissemination of findings. Their engagement is aimed at ensuring the research is responsive to each context and to facilitate the translation of findings into practice. [86]

Ethics and dissemination

The study protocol has been approved by the Royal Children's Hospital Human Research Ethics Committee (HREC/ 62866/RCHM-2020) and Sydney Local Health District (HREC/ 62866/RCHM-2020). All participants will be asked to provide written or verbal informed consent prior to completing the surveys and interviews. Each participant will be allocated a participant identification number that is kept securely under password protection accessible only to the research team.

Confidentiality

Participant confidentiality is strictly held in trust by the participating investigators, research staff, and the sponsoring institution and their agents. The study protocol, documentation, data and all other information generated will be held in strict confidence. No information concerning the study or the data will be released to any unauthorised third party other than MCRI, without prior approval by the participant.

For peer review only - http://bmjopen.bmj.com/site/about/guidelines.xhtml

Access to data

All investigators will be able to access cleaned study data for analysis. Data will be housed on Murdoch Children's Research Institute's secure network or via file transfer, and all datasets will be password protected. To ensure confidentiality, dispersed data files will not include identifying participant information.

Managing participant distress

While we do not anticipate any major risks to participants, the surveys and individual interviews may raise issues that are uncomfortable, upsetting, or frustrating for some participants. To reduce the potential for distress to participants, the study team will provide clear explanations about why the study is being conducted, how the information will be used, and the kinds of questions that will be asked. If the participant becomes distressed at any point, the researchers will empathise with the participant, stop the survey or interview if requested, and discuss ways in which they might get support from family, friends or formal support services.

Dissemination

We will publish study findings in international peer-reviewed journals and present papers at national and international conferences. At a local level in Wyndham Vale and Marrickville, we will communicate project learnings to local stakeholders via presentations, community social media, and research summaries. We will provide research summaries to local and statewide media and in social media posts. A knowledge translation strategy will disseminate findings using a range of mediums including statewide and national Hub networks, communities of practice and regular engagements with relevant government departments. The strategy is intended to support the scale up of effective components to other community health services in Victoria, NSW and across Australia.

Figure 1 Evaluation processes and phases

Figure 2. Initial Hub program theory from a realist perspective

Figure 3. Logic model proposing how the Hub models will be investigated.

Figure 4. Caregiver participant study flow

For peer review only - http://bmjopen.bmj.com/site/about/guidelines.xhtml

Acknowledgements

We thank all families, practitioners and researchers who are working together on the Child and Family Hubs project, and members of the advisory and implementation groups at each site for their oversight and guidance. We acknowledge Professor Anthony Jorm as the Chief Investigator responsible for the first stream of work (Theme A) conducted by the CRE Childhood Adversity and Mental Health.

Full references

1. Moore T, Arefadib N, Deery A, et al. The first thousand days: An evidence paper - summary. Parkville, Victoria: Centre for Community Child health, Murdoch Children's Research Institute, 2017.
2. Karatekin C, Hill M. Expanding the original definition of adverse childhood experiences (ACEs). *Journal of Child & Adolescent Trauma* 2019;12(3):289-306.
3. Lawrence D, Hafekost J, Johnson SE, et al. Key findings from the second Australian child and Adolescent Survey of Mental Health and Wellbeing. *Australian & New Zealand Journal of Psychiatry* 2016;50(9):876-86.
4. McLaughlin KA. Future directions in childhood adversity and youth psychopathology. *Journal of Clinical Child & Adolescent Psychology* 2016;45(3):361-82.
5. Jorm AF, Patten SB, Brugha TS, et al. Has increased provision of treatment reduced the prevalence of common mental disorders? Review of the evidence from four countries. *World Psychiatry* 2017;16(1):90-99.
6. Jorm AF. Why hasn't the mental health of Australians improved? The need for a national prevention strategy. *Aust N Z J Psychiatry* 2014;48(9):795-801. doi: 10.1177/0004867414546387 [published Online First: 2014/08/26]
7. Kessler RC, Angermeyer M, Anthony JC, et al. Lifetime prevalence and age-of-onset distributions of mental disorders in the World Health Organization's World Mental Health Survey Initiative. *World psychiatry* 2007;6(3):168.
8. Sahle BW, Reavley NJ, Li W, et al. The association between adverse childhood experiences and common mental disorders and suicidality: an umbrella review of systematic reviews and meta-analyses. *Eur Child Adolesc Psychiatry* 2021 doi: 10.1007/s00787-021-01745-2 [published Online First: 2021/02/28]
9. Hughes K, Bellis MA, Hardcastle KA, et al. The effect of multiple adverse childhood experiences on health: a systematic review and meta-analysis. *Lancet Public Health*

For peer review only - http://bmjopen.bmj.com/site/about/guidelines.xhtml

2017;2(8):e356-e66. doi: 10.1016/s2468-2667(17)30118-4 [published Online First: 2017/12/19]

10. Shonkoff JP, Garner AS, Siegel BS, et al. The Lifelong Effects of Early Childhood Adversity and Toxic Stress. *Pediatrics* 2012;129(1):e232-e46. doi: 10.1542/peds.2011-2663

11. Duchesne S, Vitaro F, Larose S, et al. Trajectories of Anxiety During Elementary-school Years and the Prediction of High School Noncompletion. *Journal of Youth and Adolescence* 2008;37(9):1134-46. doi: 10.1007/s10964-007-9224-0

12. Albaek AU, Kinn LG, Milde AM. Walking Children Through a Minefield: How Professionals Experience Exploring Adverse Childhood Experiences. *Qual Health Res* 2018;28(2):231-44. doi: 10.1177/1049732317734828 [published Online First: 2017/10/20]

13. Pearce J, Murray C, Larkin W. Childhood adversity and trauma: experiences of professionals trained to routinely enquire about childhood adversity. *Heliyon* 2019;5(7):e01900-e00. doi: 10.1016/j.heliyon.2019.e01900

14. Eismann EA, Theuerling J, Maguire S, et al. Integration of the Safe Environment for Every Kid (SEEK) Model Across Primary Care Settings. *Clin Pediatr (Phila)* 2019;58(2):166-76. doi: 10.1177/0009922818809481 [published Online First: 2018/10/30]

15. George S, Daniels K, Fioratou E. A qualitative study into the perceived barriers of accessing healthcare among a vulnerable population involved with a community centre in Romania. *International Journal for Equity in Health* 2018;17(1):41. doi: 10.1186/s12939-018-0753-9

16. Brown A, Rice SM, Rickwood DJ, et al. Systematic review of barriers and facilitators to accessing and engaging with mental health care among at-risk young people. *Asia Pac Psychiatry* 2016;8(1):3-22. doi: 10.1111/appy.12199 [published Online First: 2015/08/05]

17. Carrillo JE, Carrillo VA, Perez HR, et al. Defining and targeting health care access barriers. *J Health Care Poor Underserved* 2011;22(2):562-75. doi: 10.1353/hpu.2011.0037 [published Online First: 2011/05/10]

18. Alcalá HE, Valdez-Dadia A, von Ehrenstein OS. Adverse childhood experiences and access and utilization of health care. *Journal of Public Health* 2017;40(4):684-92. doi: 10.1093/pubmed/fdx155

19. Koball AM, Rasmussen C, Olson-Dorff D, et al. The relationship between adverse childhood experiences, healthcare utilization, cost of care and medical comorbidities.

For peer review only - http://bmjopen.bmj.com/site/about/guidelines.xhtml

Child Abuse & Neglect 2019;90:120-26. doi: https://doi.org/10.1016/j.chiabu.2019.01.021

20. Leutz WN. Five laws for integrating medical and social services: lessons from the United States and the United Kingdom. *Milbank Q* 1999;77(1):77-110, iv-v. doi: 10.1111/1468-0009.00125 [published Online First: 1999/04/10]
21. Edwards K, Rimes T, Smith R, et al. "Improving Access to Early Childhood Developmental Surveillance for Children from Culturally and Linguistically Diverse (CALD) Background". *Int J Integr Care* 2020;20(2):3. doi: 10.5334/ijic.4696 [published Online First: 2020/04/30]
22. Edwards K, Fernandez R, Rimes T, et al. Happy, healthy, ready-working with early childhood non-government organisations for developmental surveillance for vulnerable children. *Australian Journal of Advanced Nursing, The* 2020;37(4):37-46.
23. Yonek J, Lee CM, Harrison A, et al. Key Components of Effective Pediatric Integrated Mental Health Care Models: A Systematic Review. *JAMA Pediatr* 2020;174(5):487-98. doi: 10.1001/jamapediatrics.2020.0023 [published Online First: 2020/03/10]
24. Asarnow JR, Rozenman M, Wiblin J, et al. Integrated Medical-Behavioral Care Compared With Usual Primary Care for Child and Adolescent Behavioral Health: A Meta-analysis. *JAMA Pediatr* 2015;169(10):929-37. doi: 10.1001/jamapediatrics.2015.1141 [published Online First: 2015/08/11]
25. Lane WG, Dubowitz H, Frick KD, et al. Cost effectiveness of SEEK: A primary care-based child maltreatment prevention model. *Child Abuse Negl* 2021;111:104809. doi: 10.1016/j.chiabu.2020.104809 [published Online First: 2020/11/19]
26. Satherley R-M, Lingam R, Green J, et al. Integrated health Services for Children: a qualitative study of family perspectives. *BMC Health Services Research* 2021;21(1):167. doi: 10.1186/s12913-021-06141-9
27. Elrod JK, Fortenberry JL. The hub-and-spoke organization design: an avenue for serving patients well. *BMC Health Services Research* 2017;17(1):457. doi: 10.1186/s12913-017-2341-x
28. Malachowski C, Skopyk S, Toth K, et al. The Integrated Health Hub (IHH) Model: The Evolution of a Community Based Primary Care and Mental Health Centre. *Community Ment Health J* 2019;55(4):578-88. doi: 10.1007/s10597-018-0339-4 [published Online First: 2018/10/03]
29. Eastwood J, Barmaky S, Hansen S, et al. Refining Program Theory for a Place-Based Integrated Care Initiative in Sydney, Australia. *Int J Integr Care* 2020;20(3):13. doi: 10.5334/ijic.5422 [published Online First: 2020/10/13]

For peer review only - http://bmjopen.bmj.com/site/about/guidelines.xhtml

30. Eastwood JG, Dewhurst S, Hansen S, et al. Care Coordination for Vulnerable Families in the Sydney Local Health District: What Works for Whom, under What Circumstances, and Why? *Int J Integr Care* 2020;20(4):22. doi: 10.5334/ijic.5437 [published Online First: 2020/12/19]
31. State of Victoria. Royal Commission into Victoria's Mental Health System, Final Report, Summary and recommendations. Melbourne, Victoria: State of Victoria, 2021.
32. Australian Government National Mental Health Commission. National Children's Mental Health and Wellbeing Strategy (draft). Canberra: Australian Government, 2021.
33. Cheek JA, Craig SS, West A, et al. Emergency department utilisation by vulnerable paediatric populations during the COVID-19 pandemic. *Emerg Med Australas* 2020;32(5):870-71. doi: 10.1111/1742-6723.13598 [published Online First: 2020/07/25]
34. Australian Early Development Census. Wyndham Community 2018 [Available from: https://www.aedc.gov.au/data/data-explorer?id=135329 accessed 30/03 2022.
35. von Elm E, Altman DG, Egger M, et al. Strengthening the Reporting of Observational Studies in Epidemiology (STROBE) statement: guidelines for reporting observational studies. *Bmj* 2007;335(7624):806-8. doi: 10.1136/bmj.39335.541782.AD [published Online First: 2007/10/20]
36. Creswell JW, Plano Clark, V.L. Designing and Conducting Mixed Methods Research. Los Angeles: SAGE 2018.
37. Plattner H, Meinel C, Leifer L. Design thinking: understand–improve–apply: Springer Science & Business Media 2011.
38. Roberts JP, Fisher TR, Trowbridge MJ, et al. A design thinking framework for healthcare management and innovation. *Healthc (Amst)* 2016;4(1):11-4. doi: 10.1016/j.hjdsi.2015.12.002 [published Online First: 2016/03/24]
39. Mulvale A, Miatello A, Hackett C, et al. Applying experience-based co-design with vulnerable populations: Lessons from a systematic review of methods to involve patients, families and service providers in child and youth mental health service improvement. *Patient Experience Journal* 2016;3(1):117-29.
40. Reed JE, Green S, Howe C. Translating evidence in complex systems: a comparative review of implementation and improvement frameworks. *International Journal for Quality in Health Care* 2019;31(3):173-82. doi: 10.1093/intqhc/mzy158
41. Chambers DA, Glasgow RE, Stange KC. The dynamic sustainability framework: addressing the paradox of sustainment amid ongoing change. *Implementation Science* 2013;8(1):117.

For peer review only - http://bmjopen.bmj.com/site/about/guidelines.xhtml

42. Damschroder LJ, Aron DC, Keith RE, et al. Fostering implementation of health services research findings into practice: a consolidated framework for advancing implementation science. *Implement Sci* 2009;4:50. doi: 10.1186/1748-5908-4-50 [published Online First: 2009/08/12]
43. Langley GJ, Moen RD, Nolan KM, et al. The improvement guide: a practical approach to enhancing organizational performance: John Wiley & Sons 2009.
44. Blamey A, Mackenzie M. Theories of Change and Realistic Evaluation. *Evaluation* 2016;13(4):439-55. doi: 10.1177/1356389007082129
45. Okamura K. Interdisciplinarity revisited: evidence for research impact and dynamism. *Palgrave Communications* 2019;5(1):141. doi: 10.1057/s41599-019-0352-4
46. Pawson R, Tilley, N.,. Realistic Evaluation. London, United Kingdom: SAGE Publications 1997.
47. Wong G, Westhorp G, Manzano A, et al. RAMESES II reporting standards for realist evaluations. *BMC medicine* 2016;14(1):96.
48. Greenhalgh T, Wong G, Jagosh J, et al. Protocol—the RAMESES II study: developing guidance and reporting standards for realist evaluation. *BMJ open* 2015;5(8):e008567.
49. Nurjono M, Shrestha P, Lee A, et al. Realist evaluation of a complex integrated care programme: protocol for a mixed methods study. *BMJ Open* 2018;8(3):e017111. doi: 10.1136/bmjopen-2017-017111 [published Online First: 2018/03/04]
50. Marchal B, van Belle S, van Olmen J, et al. Is realist evaluation keeping its promise? A review of published empirical studies in the field of health systems research. *Evaluation* 2012;18(2):192-212. doi: 10.1177/1356389012442444
51. Porter S. Realist evaluation: an immanent critique. *Nursing Philosophy* 2015;16(4):239-51.
52. Bhaskar R. The possibility of naturalism: A philosophical critique of the contemporary human sciences. Florence KY, USA: Routledge 1998.
53. Archer MS, Archer MS. Realist social theory: The morphogenetic approach: Cambridge university press 1995.
54. Lemire S, Kwako A, Nielsen SB, et al. What is this thing called a mechanism? Findings from a review of realist evaluations. *New Directions for Evaluation* 2020;2020(167):73-86.
55. Hinds K, Dickson K. Realist synthesis: a critique and an alternative. *Journal of Critical Realism* 2021;20(1):1-17. doi: 10.1080/14767430.2020.1860425

For peer review only

For peer review only - http://bmjopen.bmj.com/site/about/guidelines.xhtml

56. Eastwood JG, De Souza DE, Shaw M, et al. Designing Initiatives for Vulnerable Families: From Theory to Design in Sydney, Australia. *Int J Integr Care* 2019;19(3):9. doi: 10.5334/ijic.3963 [published Online First: 2019/08/02]

57. Eastwood JG, Kemp LA, Garg P, et al. A Critical Realist Translational Social Epidemiology Protocol for Concretising and Contextualising a "Theory of Neighbourhood Context, Stress, Depression, and the Developmental Origins of Health and Disease (DOHaD)", Sydney Australia. *Int J Integr Care* 2019;19(3):8. doi: 10.5334/ijic.3962 [published Online First: 2019/08/02]

58. Eastwood JG, Woolfenden S, Miller E, et al. Implementation, Mechanisms of Effect and Context of an Integrated Care Intervention for Vulnerable Families in Central Sydney Australia: A Research and Evaluation Protocol. *Int J Integr Care* 2019;19(3):11. doi: 10.5334/ijic.4217 [published Online First: 2019/08/02]

59. Tyler I, Lynam J, O'Campo P, et al. It takes a village: a realist synthesis of social pediatrics program. *Int J Public Health* 2019;64(5):691-701. doi: 10.1007/s00038-018-1190-7 [published Online First: 2018/12/26]

60. Cantrill J, Sibbald B, Buetow S. The Delphi and nominal group techniques in health services research. *International Journal of pharmacy practice* 1996;4(2):67-74.

61. Moore T, Yagnik, Halloran, et al. Developing the Parent Engagement Resource: a tool for enhancing parent-professional relationships and identifying psychosocial issues in families. *Australian Journal of Child and Family Health Nursing* 2012;9:12-16.

62. Price A, Bryson H, Smith A, et al. Processes for engaging and retaining women who are experiencing adversity in longitudinal health research. *BMC health services research* 2019;19(1):1-12.

63. Harris PA, Taylor, R., Thielke, R., Payne, J., Gonzalez, N., Conde, J.G., . Research electronic data capture (REDCap) - A metadata-driven methodology and workflow process for providing translational research informatics support. *J Biomed Inform* 2009;42(2):377-81.

64. Mukumbang FC, Marchal B, Van Belle S, et al. Using the realist interview approach to maintain theoretical awareness in realist studies. *Qualitative Research* 2019;20(4):485-515. doi: 10.1177/1468794119881985

65. Sanson A, Prior, M., Garino, E., Oberklaid, F., Sewell, J. The Structure of Infant Temperament: Factor Analysis of the Revised Infant Temperament Questionnaire. *Infant Behavior and Development* 1987;10:97-104.

66. Squires J, Bricker D, Twombly E. The ASQ: SE user's guide: For the Ages & Stages Questionnaires: Social-emotional: Paul H Brookes Publishing 2002.

For peer review only - http://bmjopen.bmj.com/site/about/guidelines.xhtml

67. Goodman R. The Strengths and Difficulties Questionnaire: a research note. *Journal of child psychology and psychiatry* 1997;38(5):581-86.

68. Waters E, Salmon L, Wake M, et al. The Child Health Questionnaire in Australia: reliability, validity and population means. *Australian and New Zealand journal of public health* 2000;24(2):207-10.

69. Kessler RC, Barker, P.R., Colpe, L.J., Epstein, J.F., Gfroerer, J.C., Hiripi, E., Howes, M.J, Normand, S-L.T., Manderscheid, R.W., Walters, E.E., Zaslavsky, A.M.,. Screening for serious mental illness in the general population. *Archives of General Psychiatry* 2003;60(2):184-89.

70. Ware JE, Kosinski M, Keller SD. A 12-Item Short-Form Health Survey: Construction of Scales and Preliminary Tests of Reliability and Validity. *Medical Care* 1996;34(3):220-33.

71. Zubrick S, Lucas N, Westrupp E, et al. Parenting measures in the Longitudinal Study of Australian Children: construct validity and measurement quality, Waves 1 to 4. Canberra: Department of Social Services 2014.

72. EuroQol. EQ-HWB: EuroQol; 2021 [Available from: https://euroqol.org/blog/eq-hwb/ accessed 27/05 2021.

73. Australian Bureau of Statistics (ABS). Patient Experience Survey Canberra: ABS; 2010 [Available from: https://www.abs.gov.au/statistics/health/health-services/patient-experiences-australia-summary-findings accessed 02/06 2021.

74. International Wellbeing Group. Personal Wellbeing Index: . 5th Edition ed. Melbourne: Australian Centre on Quality of Life, Deakin University, 2013.

75. Lewis JM. A network approach for researching partnerships in health. *Aust New Zealand Health Policy* 2005;2:22. doi: 10.1186/1743-8462-2-22

76. Provan K, Nakama, L., Veazie, MA.,Teufel-Shone, NI., & Huddleston, C.,. Building Community Capacity Around Chronic Disease Services Through a Collaborative Interorganizational Network. *Health Education & Behavior* 2003;30(6):646-62.

77. Provan K, Harvey, J., Guernsey de Zapien, J., . Network structure and attitudes toward collaboration in a community partnership for diabetes control on the US-Mexico border. *Journal of Health Organization and Management* 2005;19(6):504.

78. Valente TW, Pitts SR. An Appraisal of Social Network Theory and Analysis as Applied to Public Health: Challenges and Opportunities. *Annu Rev Public Health* 2016 doi: 10.1146/annurev-publhealth-031816-044528

79. StataCorp. Stata Statistical Software: Release 17. Texas, US: StataCorp LLC, 2021.

For peer review only - http://bmjopen.bmj.com/site/about/guidelines.xhtml

80. R Core Team. R: A language and environment for statistical computing. Vienna, Austria: R Foundation for Statistical Computing, 2020.

81. Borgatti SP, Everett, M.G., Freeman, L.C.,. Ucinet for Windows: Software for Social Network Analysis. Harvard, MA: Analytic Technologies, 2002.

82. NVivo qualitative data analysis software [program]. Release 1.0 version, 2020.

83. Braun V, Clarke V. Reflecting on reflexive thematic analysis. *Qualitative Research in Sport, Exercise and Health* 2019;11(4):589-97. doi: 10.1080/2159676X.2019.1628806

84. Patton M. Enhancing the Quality and Credibility of Qualitative Analysis. *Health Services Research* 1999;34(5):1189-208.

85. Fielding NG. Triangulation and Mixed Methods Designs: Data Integration With New Research Technologies. *Journal of Mixed Methods Research* 2012;6(2):124-36. doi: 10.1177/1558689812437101

86. Boaz A, Hanney S, Borst R, et al. How to engage stakeholders in research: design principles to support improvement. *Health Res Policy Syst* 2018;16(1):60. doi: 10.1186/s12961-018-0337-6 [published Online First: 2018/07/13]

Authors contributions

HH, SG and JE are Chief Investigators responsible for conceptual development, study design and manuscript preparation. TH was involved in concept development, study design and drafted the manuscript. HL, SH, HML, DS, CB, AR, MY, VE, RH, LS, JF, JE, FM, SL, RJ, LC, SF, ZM, AM, GP and KD were involved in concept development, study design and manuscript preparation, and approved the final manuscript.

Funding statement

This research is supported by the Australian National Health and Medical Research Council and Beyond Blue grant number 1153419. Murdoch Children's Research Institute is supported by the Victorian Government's Operational Infrastructure Support Program. Professor Harriet Hiscock is supported by an NHMRC Practitioner Fellowship 1136222. Professor Sharon Goldfeld is supported by an NHMRC Practitioner Fellowship 1155290. Professor Jane Fisher holds the Finkel Chair in Global Health which is supported by the Finkel Family Foundation.

For peer review only - http://bmjopen.bmj.com/site/about/guidelines.xhtml

Data Availability Statement

Participant-level data will not be available upon request. Group-level data will only be available for future research for participants who endorse an optional consent for this to occur. The study does not request participant consent to share individual-level data. Participants may endorse: "Do you give your consent for your information to be kept for future research?" Group-level data will be released to approved researchers who request the data from Prof. Harriet Hiscock for studies or secondary analyses that have been approved by an Ethics Committee. The data will be available for up to 5 years following the publication of the study and will include outcome measures and baseline characteristics. The data sharing plan is available in Supplementary File 2.

Competing interests statement

There are no competing interests to declare.

Patient consent for publication

Not required.

For peer review only

Phase 1: Develop initial Hub theory (Oct 2020 – Aug 2021)

- Historical and current state needs assessment through online group discussions, interviews and meetings with intersectoral practitioners and caregivers
- Review and selection of focus CIMO configurations
- Co-design workshops and consultations to develop the Hub model

Phase 2a: Test initial Hub theory (Aug 2021 – Dec 2023)

Design: Convergent mixed-methods

Implementation, Context and Mechanism – data sources

- Caregiver baseline survey
- Hub practitioner baseline survey
- Process evaluation through routine data sources, observation, and Plan Do Study Act (PDSA) cycles
- In-depth interviews with Hub practitioners and caregivers

Outcomes

- Caregiver outcomes survey
- Hub practitioner outcomes survey
- In-depth interviews with Hub practitioners and caregivers
- Cost outcomes

Analytical approach

- Integrate data on context, mechanism and outcomes across multiple data sources
- Interpret outcomes data in reference to context and mechanism process data

Phase 3: Refine Hub model through local knowledge translation (Dec 2022-Dec 2023)

- Validate Hub theory through ongoing knowledge translation activities (e.g., forums, workshops, webinars)
- Refine Hub model based on study findings

Figure 1 Evaluation processes and phases

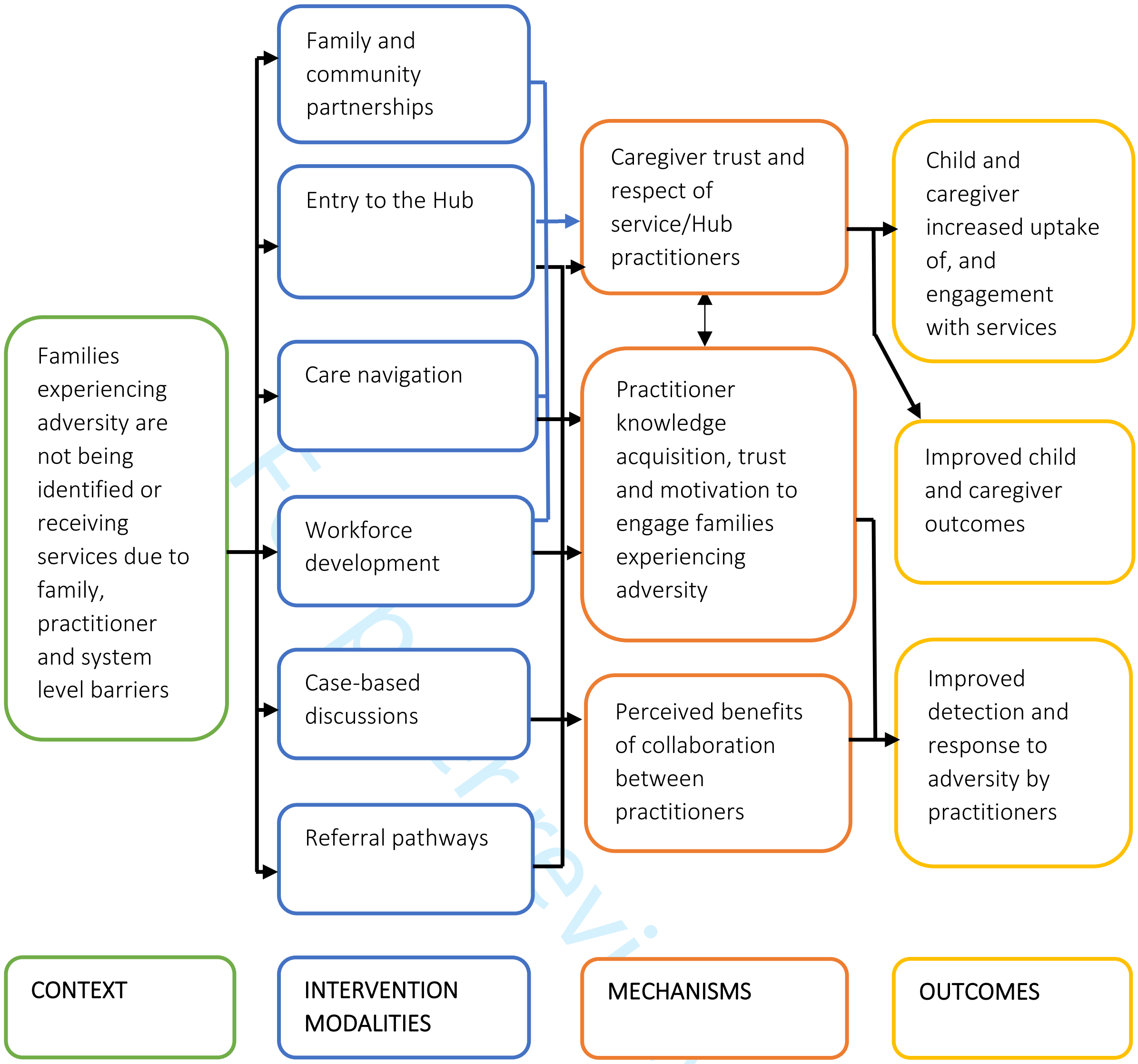

Figure 2. Initial Hub program theory from a realist perspective

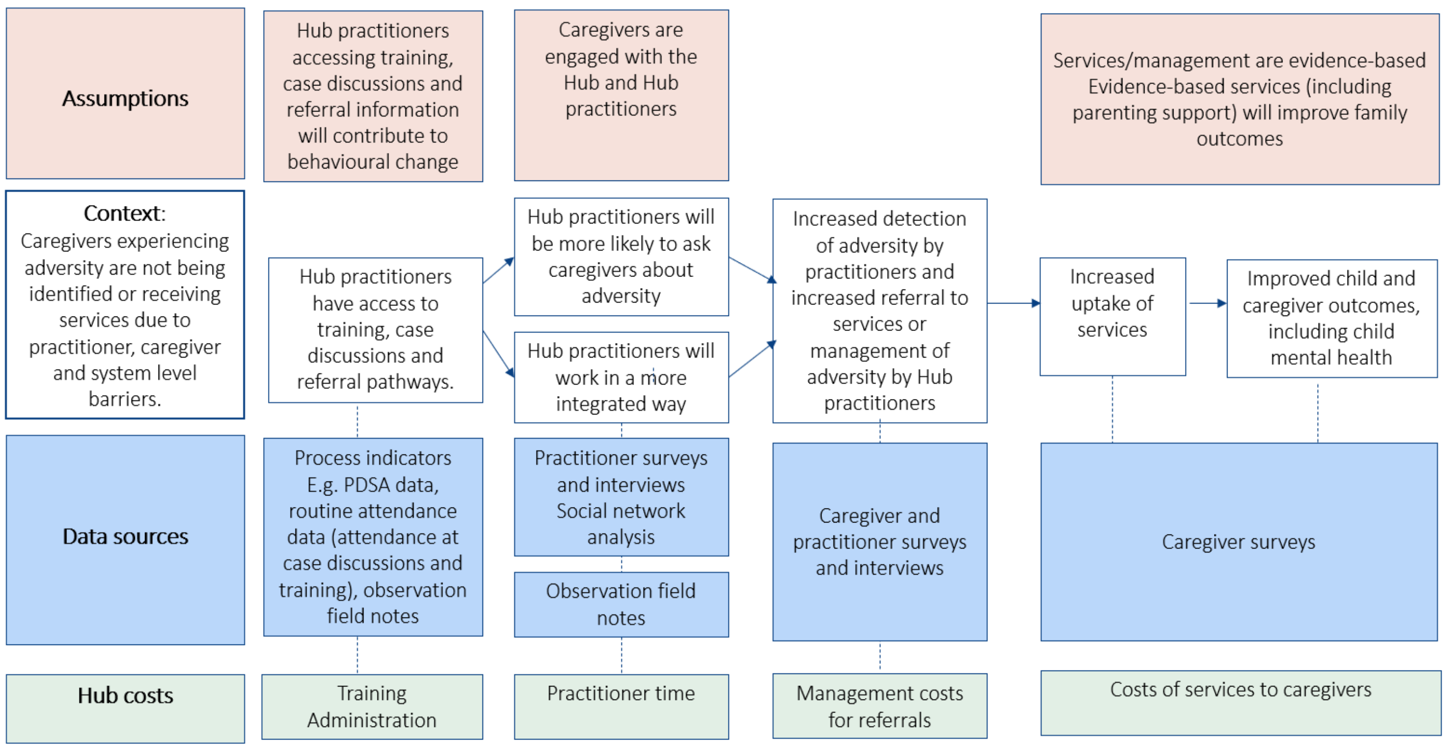

Figure 3. Logic model proposing how the Hub models will be investigated.

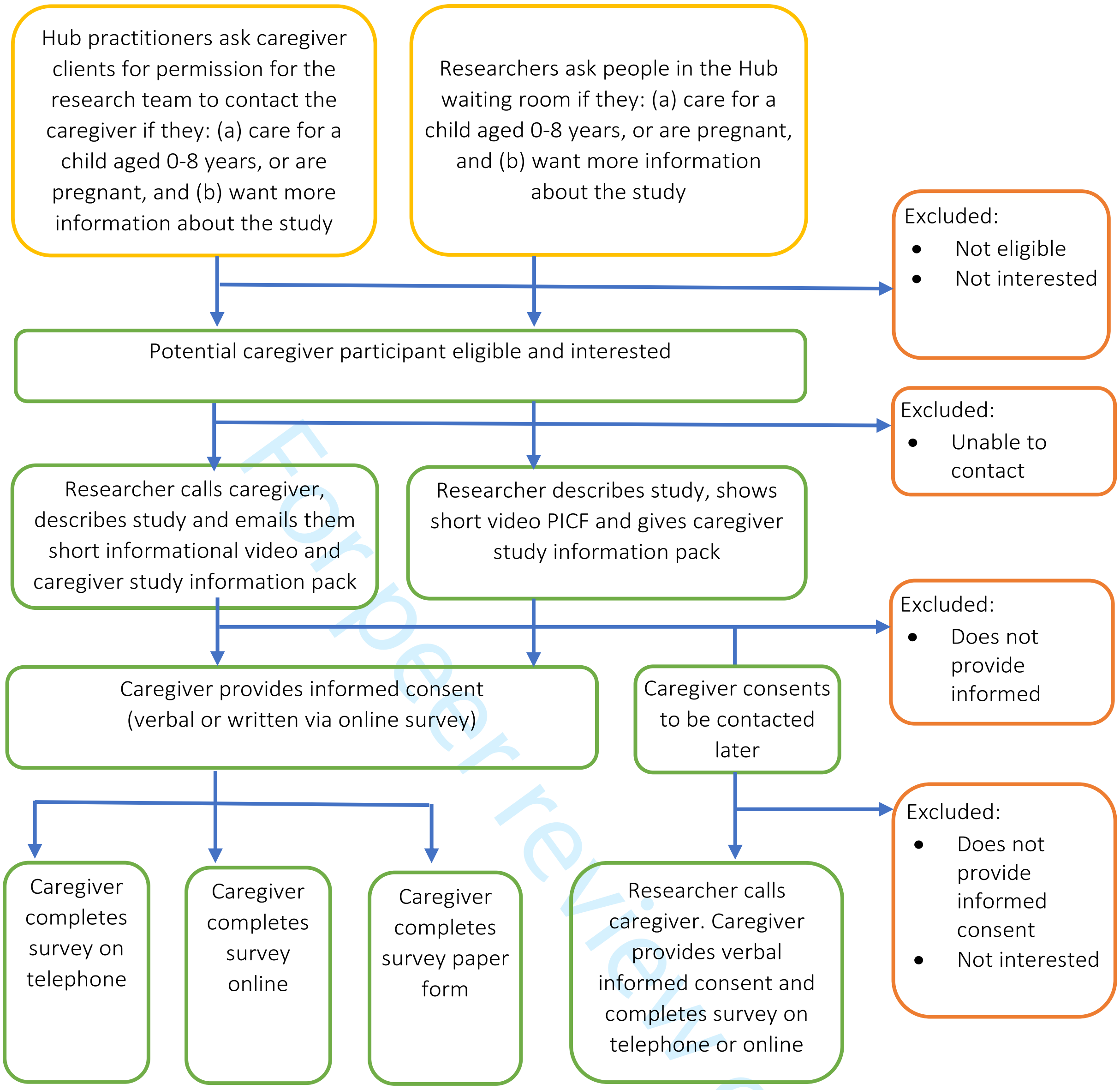

For peer review only

Child and Family Hub

CAREGIVER SURVEY

2-8 Year Old Child

ABOUT YOU AND YOUR FAMILY

Are you the main caregiver to any child aged 0-8 years or currently pregnant? Yes ☐ No ☐

How many children live in your household? ___________

Do you have any children with a disability? Yes ☐ No ☐

Do you live with another adult such as a partner or other relative? Yes ☐ No ☐

If yes, who do you live with? (Select all that apply)

☐ Partner/husband/wife ☐ Grown up children ☐ Other, please specify

☐ Parents or in-laws ☐ Other relatives

What is your year of birth? ___________

What is your gender? (Pick which best applies)

☐ Woman ☐ Non-binary/gender diverse ☐ Prefer not to say

☐ Man ☐ My gender identity isn't listed, please specify

Do you identify as Aboriginal and/or Torres Strait Islander? Yes ☐ No ☐

Where were you born? Australia ☐ Other, please specify which country ☐ ___________

What language do you mainly speak at home? English ☐ Other, please specify ☐ ___________

What was the highest year of primary or secondary school you completed? (e.g. grade 6, year 10)

____

What is your postcode? ___________

For peer review only - http://bmjopen.bmj.com/site/about/guidelines.xhtml

CHALLENGES IN LIFE

In the next three sections, there are three lists of challenges that can make it difficult for families to thrive. We will ask you *how many* of these challenges have affected your family. You do not need to tell us *which challenges*.

If you have a pen handy, it may help you to make a mark on a piece of paper for each challenge that applies to you. You can then count these up at the end. You can also count on your hands.

LIST ONE: OUTSIDE OF THE FAMILY

Over the past 6 months, how many of the following *four* challenges have you felt concerned about affecting your child/ren or family:

- Not enough contact with or support from others for yourself
- Not enough money for everyday things such as food, clothing or bills
- Problems with housing like worrying about keeping your home, having to share your home, or having a house that's too crowded, or in need of repair

☐ 1 ☐ 2 ☐ 3 ☐ 4

Thinking about the *four* challenges we just spoke about, regardless of whether or not you have been concerned about them:

In the past 6 months, has any staff member at IPC Health asked you about any of these challenges?

Yes ☐ No ☐ I don't remember ☐

If yes:

Which staff member(s)? (select all that apply)

☐ Paediatricians
☐ GPs
☐ Maternal Child Health Nurses
☐ Other Nurses
☐ Lawyers
☐ Speech Pathologists
☐ Family Violence Workers
☐ Wellbeing Coordinators
☐ Clinical Child Psychologists
☐ Family Services Workers
☐ I am not sure

Did the staff member spend extra time with you talking about or working through the challenges?

☐ Yes ☐ No ☐ I don't remember

Did the staff member connect you to a different service or organisation for support with these challenges?

☐ Yes ☐ No ☐ I don't remember

For peer review only - http://bmjopen.bmj.com/site/about/guidelines.xhtml

If yes:

Where did the staff member connect you to for support with these challenges? (select all that apply)

☐ Paediatricians
☐ GPs
☐ Maternal Child Health Nurses
☐ Other Nurses
☐ Lawyers
☐ Speech Pathologists
☐ Family Violence Workers
☐ Wellbeing Coordinators
☐ Clinical Child Psychologists
☐ Family Services Workers
☐ Someone outside of IPC Health, please specify ___________
☐ Other, not listed here, please specify ___________
☐ I am not sure

Did you go to the different service or organization?

☐ Yes, I've been
☐ Yes, I've been to some but not others
☐ No, I don't plan to go
☐ No, I'm still on a waiting list
☐ I don't remember

<u>LIST 2: INSIDE OF THE FAMILY</u>

Over the past 6 months, how many of the following *eight* challenges have you felt concerned about affecting your child/ren or family:

- My own physical health or disability or that of another family member inside or outside of my home
- My own challenging feelings like feeling emotional, depressed, angry, anxious, exhausted or even having strange thoughts such as harming myself or others
- The way that I (or my partner) manage my child/ren's daily routines, physical needs and their behaviour
- My child/ren being left to look after themselves too much, not having their needs met, or being given too much responsibility for their age
- Someone in my family drinking alcohol or using drugs
- Conflict or tension between members of my family
- My child/ren might be seeing or exposed to behaviour within the family/at home that frightens them like threats, bullying, yelling, screaming, putting people down, hitting, slapping, kicking, or punching
- Someone in the family having problems finding or keeping a job, insecure employment or a job that is not family friendly

☐ 1 ☐ 2 ☐ 3 ☐ 4 ☐ 5 ☐ 6 ☐ 7 ☐ 8

Thinking about the *eight* challenges we just spoke about, regardless of whether or not you have been concerned about them:

In the past 6 months, has any staff member at IPC Health asked you about any of these challenges?

Yes ☐ No ☐ I don't remember ☐

For peer review only - http://bmjopen.bmj.com/site/about/guidelines.xhtml

If yes:

Which staff member(s)? (select all that apply)

☐ Paediatricians
☐ GPs
☐ Maternal Child Health Nurses
☐ Other Nurses
☐ Lawyers
☐ Speech Pathologists
☐ Family Violence Workers
☐ Wellbeing Coordinators
☐ Clinical Child Psychologists
☐ Family Services Workers
☐ I am not sure

Did the staff member spend extra time with you talking about or working through the challenges?

☐ Yes ☐ No ☐ I don't remember

Did the staff member connect you to a different service or organisation for support with these challenges?

☐ Yes ☐ No ☐ I don't remember

If yes:

Where did the staff member connect you to for support with these challenges? (select all that apply)

☐ Paediatricians
☐ GPs
☐ Maternal Child Health Nurses
☐ Other Nurses
☐ Lawyers
☐ Speech Pathologists
☐ Family Violence Workers
☐ Wellbeing Coordinators
☐ Clinical Child Psychologists
☐ Family Services Workers
☐ Someone outside of IPC Health, please specify __________
☐ Other, not listed here, please specify __________
☐ I am not sure

Did you go to the different service or organization?

☐ Yes, I've been
☐ Yes, I've been to some but not others
☐ No, I don't plan to go
☐ No, I'm still on a waiting list
☐ I don't remember

<u>LIST THREE: BROADER SOCIAL NEEDS</u>

Over the past 6 months, how many of the following ***three*** challenges have you felt concerned about affecting your child/ren or family:

- Issues with visa or immigration for someone in my family or myself.
- My own or a family member's court appearances as a defendant, being on bail, parole or spending time in prison.
- Someone in the family having problems finding or keeping a job, insecure employment or a job that is not family friendly

☐ 1 ☐ 2 ☐ 3

For peer review only - http://bmjopen.bmj.com/site/about/guidelines.xhtml

Thinking about the *three* challenges we just spoke about, regardless of whether or not you have been concerned about them:

In the past 6 months, has any staff member at IPC Health asked you about any of these challenges?

Yes ☐ No ☐ I don't remember ☐

If yes:

Which staff member(s)? (select all that apply)

☐ Paediatricians
☐ GPs
☐ Maternal Child Health Nurses
☐ Other Nurses
☐ Lawyers
☐ Speech Pathologists
☐ Family Violence Workers
☐ Wellbeing Coordinators
☐ Clinical Child Psychologists
☐ Family Services Workers
☐ I am not sure

Did the staff member spend extra time with you talking about or working through the challenges?

☐ Yes ☐ No ☐ I don't remember

Did the staff member connect you to a different service or organisation for support with these challenges?

☐ Yes ☐ No ☐ I don't remember

If yes:

Where did the staff member connect you to for support with these challenges? (select all that apply)

☐ Paediatricians
☐ GPs
☐ Maternal Child Health Nurses
☐ Other Nurses
☐ Lawyers
☐ Speech Pathologists
☐ Family Violence Workers
☐ Wellbeing Coordinators
☐ Clinical Child Psychologists
☐ Family Services Workers
☐ Someone outside of IPC Health, please specify __________
☐ Other, not listed here, please specify __________
☐ I am not sure

Did you go to the different service or organization?

☐ Yes, I've been
☐ Yes, I've been to some but not others
☐ No, I don't plan to go
☐ No, I'm still on a waiting list
☐ I don't remember

For peer review only - http://bmjopen.bmj.com/site/about/guidelines.xhtml

Child and Family Hub

YOUR CHILD

What is the name and date of birth of the child aged 0-8 years old that is currently requiring the most support?

Name: ___________ Date of birth: ___________ ☐ I don't have any children, I am currently pregnant

	Excellent	Very good	Good	Fair	Poor
In general, would you say your child's health is:	☐	☐	☐	☐	☐

For each item, please mark the box for Not True, Somewhat True or Certainly True. It would help us if you answered all items as best you can even if you are not absolutely certain. Please give your answers on the basis of your child's behaviour over the last six months.

	Not true	Somewhat true	Certainly true
• Considerate of other people's feelings	☐	☐	☐
• Restless, overactive, cannot stay still for long	☐	☐	☐
• Often complains of headaches, stomach-aches or sickness	☐	☐	☐
• Shares readily with other children, for example toys, treats, pencils	☐	☐	☐
• Often loses temper	☐	☐	☐
• Rather solitary, prefers to play alone	☐	☐	☐
• Generally well behaved, usually does what adults request	☐	☐	☐
• Many worries or often seems worried	☐	☐	☐
• Helpful if someone is hurt, upset or feeling ill	☐	☐	☐
• Constantly fidgeting or squirming	☐	☐	☐
• Has at least one good friend	☐	☐	☐
• Often fights with other children or bullies them	☐	☐	☐
• Often unhappy, depressed or tearful	☐	☐	☐
• Generally liked by other children	☐	☐	☐
• Easily distracted, concentration wanders	☐	☐	☐
• Nervous or clingy in new situations, easily loses confidence	☐	☐	☐
• Kind to younger children	☐	☐	☐

For peer review only

For peer review only - http://bmjopen.bmj.com/site/about/guidelines.xhtml

• Often argumentative with adults	☐	☐	☐
• Picked on or bullied by other children	☐	☐	☐
• Often volunteers to help others (parents, teachers, other children)	☐	☐	☐
• Can stop and think things out before acting	☐	☐	☐
• Can be spiteful to others	☐	☐	☐
• Gets along better with adults than with other children	☐	☐	☐
• Many fears, easily scared	☐	☐	☐
• Good attention span, sees chores or homework through to the end	☐	☐	☐

Overall, do you think that your child has difficulties in one or more of the following areas: emotions, concentration, behaviour or being able to get on with other people?

No	Yes – minor difficulty	Yes – definite difficulties	Yes – severe difficulties
☐	☐	☐	☐

If you have answered "Yes", please answer the following questions about these difficulties:
How long have these difficulties been present?

Less than a month	1-5 months	6-12 months	Over a year
☐	☐	☐	☐

Do the difficulties upset or distress your child?

Not at all	Only a little	Quite a lot	A great deal
☐	☐	☐	☐

Do the difficulties interfere with your child's everyday life in the following areas?

	Not at all	Only a little	Quite a lot	A great deal
• Home life	☐	☐	☐	☐
• Friendships	☐	☐	☐	☐
• Learning	☐	☐	☐	☐
• Leisure activities	☐	☐	☐	☐

Do the difficulties upset or distress your child?

Not at all	Only a little	Quite a lot	A great deal
☐	☐	☐	☐

For peer review only

YOU AND YOUR LIFE

These next questions are trying to measure how your life has been over the last 7 days.

In the last 7 days:	No difficulty	Slight difficulty	Some difficulty	A lot of difficulty	Unable
• How difficult was it for you to do day-to-day activities? *(for example, working, shopping, housework*)	☐	☐	☐	☐	☐
• How difficult was it for you to get around inside and outside? *(using any aids you usually use, for example, walking stick, frame or wheelchair)*	☐	☐	☐	☐	☐

	No	Mild	Moderate	Severe	Very severe
Please select one of the following to describe your experience in the last 7 days: I had _____ physical pain	☐	☐	☐	☐	☐

In the last 7 days:	Never	Only occasionally	Sometimes	Often	Most or all of the time
• I felt lonely	☐	☐	☐	☐	☐
• I felt I had no control over my day to day life *(having the choice to do things or have things done for you as you like and when you want)*	☐	☐	☐	☐	☐
• I had trouble concentrating/thinking clearly	☐	☐	☐	☐	☐
• I felt anxious	☐	☐	☐	☐	☐
• I felt exhausted	☐	☐	☐	☐	☐
• I felt sad/depressed	☐	☐	☐	☐	☐

	Excellent	Very good	Good	Fair	Poor
In general, would you say your own health is?	☐	☐	☐	☐	☐

For peer review only

The next set of questions ask about your experience with the Child and Family Hub@ IPC Health Wyndham Vale.

The Child and Family Hub@ IPC Health Wyndham Vale includes the following practitioners:

- GPs
- Paediatricians
- Nurses (including maternal child health nurses, GP practice nurses and other nurses)
- Child speech pathologists
- Child psychologists
- Wellbeing coordinators
- Lawyers

For peer review only - http://bmjopen.bmj.com/site/about/guidelines.xhtml

Thinking about all the practitioners you have seen at the Child and Family Hub@ IPC Health Wyndham Vale in the last 6 months, how often did they:

	Always	Often	Rarely	Sometimes	Never
• Listen carefully to you?	☐	☐	☐	☐	☐
• Show respect for what you had to say?	☐	☐	☐	☐	☐
• Spend enough time with you?	☐	☐	☐	☐	☐

Would you recommend Child and Family Hub@ IPC Health Wyndham Vale to other families?

☐ Yes ☐ No

The next set of questions ask you about your relationship with your child.

If you have more than one child, please answer about the child that is currently requiring the most support.

	Never/ Almost never	Rarely	Sometimes	Often	Always/ Almost always
• How often do you express affection by hugging, kissing and holding your child?	☐	☐	☐	☐	☐
• How often do you hug or hold your child for no particular reason?	☐	☐	☐	☐	☐
• How often do you tell your child how happy he/she makes you?	☐	☐	☐	☐	☐
• How often do you have warm, close times together with your child?	☐	☐	☐	☐	☐
• How often do you enjoy listening to your child and doing things with him/her?	☐	☐	☐	☐	☐
• How often do you feel close to your child both when he/she is happy and upset	☐	☐	☐	☐	☐
• Does your child behave in a manner different from the way you want him/her to?	☐	☐	☐	☐	☐
• Do you think that your child's behaviour is more than you can handle?	☐	☐	☐	☐	☐
• Do you feel that you are good at getting your child to do what you want him/her to do?	☐	☐	☐	☐	☐
• Do you feel that you are in control and on top of things when you are caring for your child?	☐	☐	☐	☐	☐
• I have been angry with my child?	☐	☐	☐	☐	☐
• I have raised my voice with or shouted at my child?	☐	☐	☐	☐	☐
• When my child cries, he/she gets on my nerves?	☐	☐	☐	☐	☐
• I have lost my temper with my child?	☐	☐	☐	☐	☐
• I have left my child alone in his/her bedroom when he/she was particularly upset?	☐	☐	☐	☐	☐

For peer review only

For peer review only - http://bmjopen.bmj.com/site/about/guidelines.xhtml

During the past 30 day, about how often did you feel…	All of the time	Most of the time	Some of the time	A little of the time	None of the time
• …nervous?	☐	☐	☐	☐	☐
• …hopeless?	☐	☐	☐	☐	☐
• …restless or fidgety?	☐	☐	☐	☐	☐
• …so depressed that nothing could cheer you up?	☐	☐	☐	☐	☐
• …that everything was an effort?	☐	☐	☐	☐	☐
• …worthless?	☐	☐	☐	☐	☐

	No satisfaction at all									Completely Satisfied
	1	2	3	4	5	6	7	8	9	10
• How satisfied are you with your standard of living?	☐	☐	☐	☐	☐	☐	☐	☐	☐	☐
• How satisfied are you with your health?	☐	☐	☐	☐	☐	☐	☐	☐	☐	☐
• How satisfied are you with what you are achieving in life?	☐	☐	☐	☐	☐	☐	☐	☐	☐	☐
• How satisfied are you with your personal relationships?	☐	☐	☐	☐	☐	☐	☐	☐	☐	☐
• How satisfied are you with how safe you feel?	☐	☐	☐	☐	☐	☐	☐	☐	☐	☐
• How satisfied are you with feeling part of your community?										
• How satisfied are you with your future security?	☐	☐	☐	☐	☐	☐	☐	☐	☐	☐
• How satisfied are you with your spirituality or religion?	☐	☐	☐	☐	☐	☐	☐	☐	☐	☐
	☐	☐	☐	☐	☐	☐	☐	☐	☐	☐

For peer review only

For peer review only - http://bmjopen.bmj.com/site/about/guidelines.xhtml

SERVICE USAGE IN THE PAST 6 MONTHS

This next set of questions is about different services you may have used.

In the past 6 months, have you been to any of the services below to help with your child's health or wellbeing, or any of the family life challenges we have talked about today?

☐ No ☐ Yes. If yes, please tell us the number of times you went to each service and the cost to you.

	Number of times in the past 6 months	What is the cost to your family to use this service each visit? (amount you pay over and above what you get back from Medicare and private health insurance)
GP	____visits	$______
Paediatrician	____visits	$______
Maternal and Child Health Nurse	____visits	$______
School nurse	____visits	$______
Psychologist	____visits	$______
Social worker	____visits	$______
Legal support or lawyer	____visits	$______
Support service (e.g. Family violence support, alcohol and drug support, housing support or parenting support)	____visits	$______
Phone helpline (please specify):	____calls	
Other (please specify):	____	$______

Have you or your partner (if applicable) had to take any time off from usual activities (such as paid work, study, voluntary work, housekeeping) during the last 6 months because of your child's health or well-being? Please fill in below.

	Had time off paid work?	Had time off other usual activities?
Yourself	☐ No<br>☐ Yes, ____ days off<br>☐ Not applicable	☐ No<br>☐ Yes, ____ days off<br>☐ Not applicable
Your partner	☐ No<br>☐ Yes, ____ days off<br>☐ Not applicable	☐ No<br>☐ Yes, ____ days off<br>☐ Not applicable

For peer review only - http://bmjopen.bmj.com/site/about/guidelines.xhtml

Child and Family Hub

Thank you for your time completing this survey today. Your answers will be helpful to allow us to see if the Hub is working to better support families!

😊

For peer review only

For peer review only - http://bmjopen.bmj.com/site/about/guidelines.xhtml
